# Using financial diaries to understand the economic lives of HIV-positive pregnant women and new mothers in PMTCT in Zomba, Malawi

**DOI:** 10.1371/journal.pone.0252083

**Published:** 2021-07-30

**Authors:** Lara Lorenzetti, Mandy Swann, Andres Martinez, Amy O’Regan, Jamilah Taylor, Alexis Hoyt

**Affiliations:** 1 Global Health and Population Research, Durham, North Carolina, United States of America; 2 Global Education, Employment, and Engagement, Washington, District of Columbia, United States of America; 1. IRCCS Neuromed 2. Doctors with Africa CUAMM, ITALY

## Abstract

**Background:**

Economic strengthening (ES) interventions can address economic barriers to retention and adherence (R&A) to antiretroviral therapy in prevention of mother-to-child transmission (PMTCT) services. To inform tailoring of ES activities for PMTCT, we used financial diaries to understand the economic lives of women in PMTCT and examine associations between participants’ finances and their R&A.

**Methods:**

We collected financial data from a stratified sample (n = 241) of HIV-positive pregnant women and new mothers enrolled in PMTCT from three clinics in Zomba, Malawi. For 30 weeks, participants met with staff to record cash and in-kind inflows and outflows. We used clinical records to calculate a measure of R&A for each participant. We summarized diary data using R and used cox proportional hazard models to examine the relationship between R&A and participant characteristics and behavior.

**Results:**

There were 68,097 cash transactions over 30 weeks, with 10% characterized as inflows. The median value of cash inflows was US$3.54 compared with US$0.42 for cash outflows. Fewer than 7% of total transactions were considered related to PMTCT, with the majority classified as food or drink. Participants in the rural site had the lowest hazard of non-adherence. Decreased hazard of non-adherence was also linked to having dependents and years on ART. There were significant differences in cash inflows and outflows between those who were always adherent and those who were not.

**Conclusions:**

Financial inflows were large and erratic, whereas outflows were small but consistent. PMTCT expenses comprised a small proportion of overall expenses and focused on proper nutrition. The influence of inflows and outflows on adherence was significant but small; however, always adherent participants demonstrated smoother inflows and outflows, indicating an association between greater adherence and economic stability. Participants would benefit from interventions that bolster and stabilize their economic lives, including income generating activities in the agricultural industry and inclusion in village banks.

## Background

In 2011, Malawi piloted Option B+, an innovative treatment approach allowing HIV-positive pregnant and breastfeeding women to immediately begin taking antiretroviral therapy (ART), regardless of CD4 count, and continue for life [1]. Infants born to these women receive daily treatment for up to six weeks after birth, at which point they are tested for HIV. Mothers are counseled to exclusively breastfeed for six months and continue breastfeeding for up to 24 months. The shift to Option B+ resulted in a dramatic increase in the number of women starting ART [1]. Moreover, Option B+ significantly increased the duration of maternal ART therapy pre-delivery, reduced HIV transmission to HIV-exposed infants, and decreased maternal mortality in the first year of treatment in Malawi [2–4]. Building on Malawi’s success, the World Health Organization (WHO) incorporated Option B+ into its international guidelines. Since 2010, the number of HIV-positive pregnant women across priority countries receiving ART has nearly doubled from 44% to an estimated 80% in 2018 [5].

Despite these laudable efforts to increase the number of HIV-positive pregnant women and new mothers (PWNM) initiating ART and enrolling into prevention of mother-to-child (PMTCT) services, retention in care and adherence to treatment (R&A) continues to present a challenge for epidemic control, including prevention of new HIV infections in children. As of 2018, the global mother-to-child transmission rate was 12% [5], owed in large part to a lack of ART coverage for HIV-positive PWNM and issues with R&A. Specifically in Malawi, approximately 20% of women enrolled in PMTCT at the largest antenatal clinic were considered lost to follow up (LTFU) [6]. Additionally, Kim et al. (2015) found that LTFU increased incrementally over the PMTCT treatment cascade [3]. An analysis of nationwide facility-level data in Malawi demonstrated that 21.6% of women were LTFU from PMTCT 12 months after treatment initiation, and 27.3% and 28.1% were LTFU at 24 and 36 months, respectively [7].

**Fig 1:**
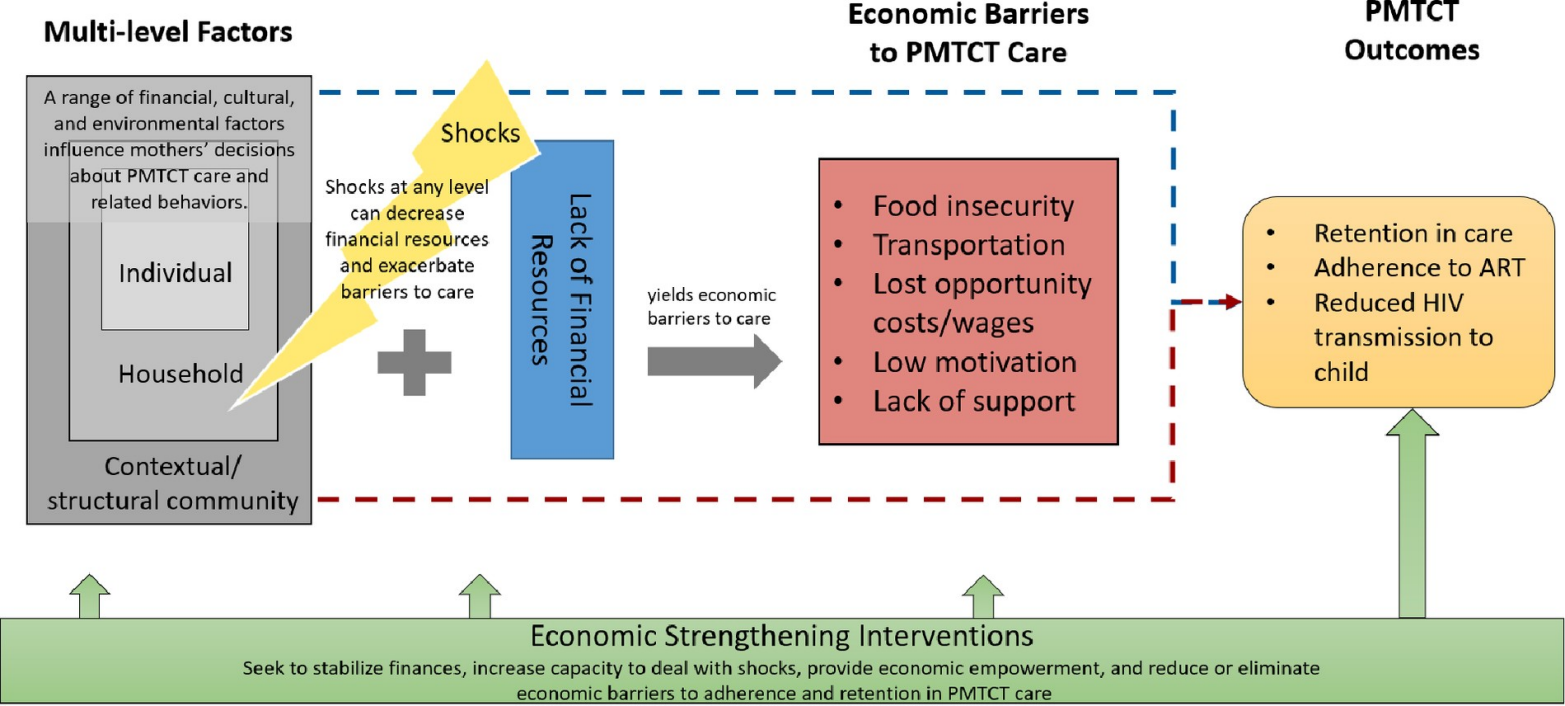
Conceptual model of economic barriers to PMTCT outcomes and the role of economic strengthening interventions.

Issues with PMTCT R&A can be linked to several economic barriers [8]. Most notably, the cost of transportation to monthly facility appointments was considered prohibitive in Malawi [8, 9], where the average cost of getting to the facility in one rural site was equivalent to the cost of food for an adult for the entire day [10]. Food insecurity is also linked to issues with R&A, since taking ART on an empty stomach can lead to severe side effects and treatment interruption in some cases. Food insecurity also undermines women’s ability to breastfeed exclusively, which can increase the risk of vertical transmission [11]. Fig 1 demonstrates how lack of financial resources can yield economic barriers to care that influence PMTCT care-seeking and treatment outcomes.

Addressing these barriers to PMTCT care requires structural interventions that complement clinical HIV services and support treatment outcomes. Economic strengthening (ES) interventions, including savings groups and income generating activities, can intervene at the personal, household, or community levels to stabilize finances, increase economic security, and improve the capacity to deal with shocks. However, few studies have focused on ES activities to increase R&A specifically for PMTCT [11]. Notably, in a randomized controlled trial in the Democratic Republic of the Congo, cash transfers and food support demonstrated some promise for improving retention in PMTCT [12]. Given the limited research in this area, it remains unclear how ES can best support PMTCT programming. Moreover, there is also a dearth of information about women’s financial lives throughout the PMTCT cascade, which further limits our understanding of how ES would support PMTCT R&A.

We conducted an exploratory study using the financial diaries (FD) methodology in three study sites in Zomba, Malawi. There were two main objectives for this study: 1) to provide a comprehensive and contextualized understanding of the financial lives of HIV-positive PWNM; and 2) to examine the relationship between financial behaviors and PMTCT R&A. These findings inform recommendations for appropriate ES interventions to support PMTCT R&A. This study is innovative in its use of FDs to understand the economic lives of a population experiencing a range of economic barriers to care and for whom the risk of transmission remains high.

## Methods

We conducted a mixed-methods exploratory study using FDs, a quantitative baseline survey, qualitative endline in-depth interviews (IDIs), and a review of clinical record data on PMTCT-related care. This study was granted ethical approval by Malawi’s National Health Sciences Research Committee and FHI 360’s Protections of Human Subjects Committee.

### Study sites

This study was conducted in three sites in Zomba, a PEPFAR-priority district in the southern region of Malawi, with a high adult HIV prevalence rate of 16.3% compared with the national average of 11.0% [13]. Within Zomba, we selected three study sites from distinct geographies. Site eligibility included having a high volume of women enrolled in PMTCT and preference for facilities using electronic medical records. Zomba’s District Health Management Team provided guidance on site selection, which ultimately included two public facilities, Likangala Health Center (rural) and Matawale Health Center (urban), and one private facility, Pirimiti Community Hospital (peri-urban). The PMTCT programs were similar across sites and followed national guidelines under Option B+. These included: HIV testing for all pregnant women; ART for all HIV-positive pregnant women irrespective of immunological status; at least four antenatal care appointments; HIV-exposed newborns given nevirapine daily from birth through six weeks; HIV testing at six weeks, 12 months, and 24 months for HIV-exposed infants; cotrimoxazole preventive therapy for newborns from six weeks through 24 months if HIV-negative; and universal ART for newborns if HIV-positive. These appointments may be in addition to medication pick-ups for maternal ART.

### Eligibility criteria

To generate financial data across the breadth of the PMTCT cascade, we used stratified random sampling to recruit HIV-positive PWNM into four sub-groups. At each site, participants were sampled from a list of women who, at baseline, were: 1) pregnant; or were ±4 weeks from being 2) 2 months post-partum; 3) 8 months post-partum; or 4) 14 months post-partum. In addition to meeting a sub-group requirement, eligibility criteria included being at least 18 years of age and being enrolled in PMTCT and initiated on ART, as confirmed by their medical record.

### Sampling & recruitment

Sampling and recruitment were conducted from June-July 2018 by a Malawian data collection firm that received study-specific and research ethics training, including special protections for working with people living with HIV. Recruitment was conducted in consort with health facility staff at each site, who created an initial de-identified listing of PMTCT participants based on their pregnancy and post-partum status. This list was shared with the local firm, who then worked with principal investigators to randomly sample from within each sub-group. In some cases, the number of potential participants per sub-group was smaller than anticipated, and in these cases, all participants were recruited.

To protect confidentiality, lists of randomly selected potential participants were shared with clinical staff and expert clients who were responsible for routine communication and follow-up with women in PMTCT. Staff and expert clients used existing forms of contact such as calls or home visits to inform women about the study and invite them to meet with the study team. Those interested in participating were screened and provided informed consent, including their approval to access information in their medical records. Once PMTCT and ART status were confirmed, women were enrolled into the study and worked with assigned data collectors to establish a weekly date, time, and secure location to complete the FD interview. A breakdown of enrolled participants is included in Table 1.

**Table 1:** Sample size and stratification.

Site	Group	Sample Size
Matawale (urban facility) (83)	Pregnant	25
	2 months post-partum	12
	8 months post-partum	24
	14 months post-partum	22
Pirimiti (peri-urban facility) (80)	Pregnant	11
	2 months post-partum	22
	8 months post-partum	27
	14 months post-partum	20
Likangala (rural facility) (78)	Pregnant	20
	2 months post-partum	11
	8 months post-partum	27
	14 months post-partum	20
	**Total**	**241**

We planned to recruit 330 participants, 110 at each site accounting for 10% attrition across the 30-week data collection period. Given that this was an exploratory study not testing an intervention, sample size estimates were based on logistical and budgetary considerations rather than on statistical power to detect specific differences between groups.

From the list of all enrolled participants, we randomly selected 45 women to participate in endline IDIs. These interviews were conducted separately from FD interviews and required a separate consent process. We sampled 48 FD participants, 16 from each site, to participate in IDIs, though three women were ultimately unable to participate.

### Data collection & tools

We collected FD data for 30 weeks, from July 2018 to February 2019. FDs are a high-frequency panel data collection approach that produces a robust dataset on participants’ cash and in-kind income and expenditures (hereafter, inflows and outflows, respectively). The FD methodology was first piloted in Bangladesh, India, and South Africa as outlined in *Portfolios of the Poor* [14] but has since been expanded on and utilized by various organizations working in low-income contexts. This method is regularly applied in resource-poor settings and among populations with a range of literacy and numeracy skills. We conducted weekly face-to-face interviews with participants to record their financial transactions. This study adapted the standard paper-based FD debriefing template into a tablet-based format, allowing for real-time data uploads and consistency checks. Using tablets also facilitated classifying transactions by type and sorting inflows and outflows into categories (Table 2).

**Table 2:** Financial diary transaction types and categories.

Transaction Types	Categories
Cash (earned income or expenditures)	Food/drink
Intra-household transfers	Household
Gifts	Child Expenses
Loans	Transportation
Debt Repayment	Communication
Savings	Medical/Health
Remittances	Agriculture
Barters (non-cash)	Discretionary

Importantly, we examined women-controlled finances––not household-level finances––which limited inflows and outflows to transactions that participants directly engaged in. Acknowledging the importance of husbands/partners in the financial lives of women, the FD included intra-household transfers (IHTs) as a transaction type. In addition to recording transaction type and category for each inflow and outflow, we also asked for information on where and with whom the transaction took place and if the transaction had a household, business, or mixed purpose. Finally, participants were asked to flag any expenditures that were related to PMTCT or general health care. English and Chichewa language versions of the FD debriefing tool are available as Supporting information.

FD data were collected for 30 weeks. Although the FD questionnaire was not validated among the study population prior to the study, the first four weeks of data collection were considered a “ramp-up” period as both participants and data collectors were becoming familiar with the methodology. This period built confidence in how to categorize responses and provide the appropriate level of detail. These four weeks were excluded from analysis. In January and February 2019, we also collected retrospective clinical data on ART pick-ups (date and number of pills dispensed) for each participant using a combination of electronic and paper records at each facility.

Participants completed quantitative baseline survey in July 2018, which included sections on: 1) basic demographic information; 2) household characteristics; 3) income sources and financial management; 4) economic shocks and benefits; 5) experience with PMTCT; 6) food security and dietary diversity; 7) decision-making and autonomy; and 8) group membership and social support. Endline IDIs were conducted using structured guides which included sections on: 1) participant experience keeping the financial diary; 2) financial behaviors and PMTCT; and 3) PMTCT decision-making. Some questions asked participants to reflect on their FD, including providing context for outlier outflows and explaining why certain expenditures were PMTCT-related. Guides for the baseline survey and endline IDIs are also available in Supporting information.

### Analysis

We collected data on cash, in-kind, and barter transactions. However, participants consistently had difficulty estimating cash values for non-cash transactions (i.e., barter and in-kind). Therefore, non-cash transactions were excluded from this analysis since their incorporation with cash data would reduce confidence in our estimates. We conducted a descriptive analysis of the FD data focused exclusively on cash transactions, where a woman bought or sold something for cash. We also descriptively analyzed data from the baseline survey using R.

#### Measures of adherence

To examine the relationship between financial behavior and R&A, we calculated two measures of adherence. First, we used ART pick-up dates and pill counts to construct a measure of the proportion of days covered (PDC), calculated as:

$$\frac{Number\;of\;days\;in\;the\;period\;that\;patient\;had\;ART\;in\;possession}{Number\;of\;days\;in\;period}\;x\;100$$

Calculating PDC for each participant required the creation of a day-level dataset indicating if a participant had a pill for each day between their scheduled pick-ups. It was common for participants to receive extra pills at each pick-up to help them stay adherent if they could not make their scheduled appointment. As a result, participants often built pill reserves. Our approach took these reserves into consideration, allowing extra pills early in the PMTCT cascade to carry over and cover them during treatment gaps that occurred later in the cascade. Despite these reserves, PDC as a measure was censored at 100%.

Using that same day-level dataset, we also constructed a binary measure of adherence indicating if a participant was adherent in any given week. Using 90% adherence as a clinically significant threshold, we considered four or more days without a pill as non-adherent for the corresponding week. Four or more days within a 30/31-day period drops a participant to less than 90% adherence for that month. Given that the four-day window could span two weeks, participants were only considered non-adherent for the week corresponding to day four and beyond. Using the binary weekly adherence measure we assessed the proportion of women in our sample who were adherent throughout the weeks of the PMTCT cascade. To understand how adherence related to cash flows, we also used a simple model regressing cash inflows and cash outflows separately on this binary adherence variable. We controlled for study week and included robust standard errors to adjust for autocorrelation.

#### Kaplan-Meier curves & Cox proportional hazard models

We used the weekly binary measure of adherence to conduct survival analyses and estimate Kaplan-Meier curves indicating the probability of time to first non-adherence within our sample, disaggregated by baseline factors. We used the chi-square statistic of the log-rank test for equality of survival functions. This provided a measure of distance between the Kaplan-Meier curves on the same plot, indicating whether there is a statistically significant difference between them. We also used the binary measure of adherence as our dependent variable in a Cox proportional hazards regression model to assess the factors that are predictive of non-adherence over the PMTCT cascade. In the Cox proportional hazard model, we included nine control variables: age, marital status, site, group, highest education, years on ART, number of direct dependents, and average cash inflows and outflows. These analyses were done in Stata.

#### IDI analysis

IDIs were audio-recorded, transcribed in Chichewa, and translated into English by the local data collection firm. All transcripts were analyzed using NVivo 12. A codebook was developed based on a reading of the translated transcripts and identifying emergent themes [15]. Two researchers coded 15% of transcripts using the codebook and conducted a coding comparison to assess inter-coder reliability and address discrepancies in interpretation and use of the codebook. Researchers then split the remaining transcripts for coding. Coded transcripts were summarized by theme to identify factors associated with R&A in PMTCT. In the results section, we used the qualitative findings to provide deeper insight into the findings from the FD data.

## Results

We recruited 241 participants across all three sites. This was considerably less than our anticipated sample of 330. The primary reason we were unable to achieve our anticipated sample size, which was estimated prior to site selection, was that there were fewer women in each of the three selected facilities who met our overall eligibility criteria as well as our sub-group criteria. For those who were eligible, the main reason for not participating was inability to contact the potential participant (i.e., made up to three contact attempts with no response). In few cases, participants were unable to commit to the 30-week study window.

### Participant characteristics

The median participant age at baseline was 30 and most participants across all sites were married. There were notable differences in schooling and ability to read between all three sites. Overall, most participants had completed primary school, but roughly 32% could not read at all. (Table 3).

**Table 3:** Baseline demographics of financial diary participants in Zomba, Malawi in July 2018.

	Likangala (Rural)	Pirimiti	Matawale	Whole sample
		(Peri-Urban)	(Urban)	
	n = 78	n = 80	n = 83	n = 241
Age (median and range)	32 (18–45)	29.5 (18–49)	31 (18–49)	30 (18–49)
Marital Status				
Married/partnered, %	68	78	78	75
Unmarried/unpartnered, %	32	22	22	25
Ever been to school, %	72	88	96	85
Highest grade completed				
Primary school, %	65	66	72	68
Lower secondary, %	4	9	8	7
Upper secondary, %	3	13	14	10
Trade school, %	0	0	0	0
University or higher, %	0	0	1	<1
Other, %	0	0	0	0
Cannot read at all, %	51	38	8	32
Able to read part of the sentence, %	14	6	33	18
Able to read the entire sentence, %	35	56	59	50
Blind/visually impaired, %	0	0	0	0
Religion				
Christian, %	87	96	70	84
Muslim, %	13	4	23	13
Traditional, %	0	0	0	0
No religion, %	0	0	0	0
Other, %	0	0	7	2
Simple Poverty Scorecard: PPI (median and range)	31 (9–91)	34 (9–65)	46 (13–95)	37 (9–95)
Children under 18 living in the household (median and range)	4 (0–7)	4 (0–7)	3 (0–6)	3 (0–7)
Direct dependents (median and range)	4 (0–7)	4 (0–7)	3 (0–6)	3 (0–7)
Worked to earn money in the past 30 days, %	69	59	57	61
Estimated income in past 30 days in kwacha (median and range)	4,250 (400–75,000)	5,000 (88–70,000)	7,000 (88–200,000)	5,750 (88–200,000)
Estimated income in past 30 days in USD (median and range)	5.90 (0.56–104.17)	6.94 (0.12–97.22)	9.72 (0.12–277.78)	7.99 (0.12–277.78)
Had a savings goal over last 30 days, %	56	61	36	51
Took loans out in the past 30 days, %	84	93	90	90
Experienced shock in the past 6 months (%)	99	99	84	94
Have you disclosed your HIV status to your husband/partner?				
Yes, %	88	88	88	88
No, %	5	4	1	3
No partner, %	6	9	11	9
Disclosed her HIV status to any other family or friends, %	78	79	81	79
Used PMTCT services for other pregnancies other than current/recent pregnancy, %	96	90	79	88
Multidimensional Scale of Perceived Social Support (median and range)	4.0 (1–5)	4.3 (1.8–5)	3.9 (1–5)	4.1 (1–5)
Significant Other Subscale (median and range)	4.3 (1–5)	4.6 (1–5)	4.3 (1–5)	4.5 (1–5)
Family Subscale (median and range)	4.5 (1–5)	4.5 (1–5)	4.5 (1–5)	4.5 (1–5)
Friends Subscale (median and range)	4.0 (1–5)	4.0 (1–5)	3.0 (1–5)	3.8 (1–5)

Fewer than two-thirds of participants had worked in the 30 days prior to baseline to earn money, with more women working in Likangala (69%) than in the other two sites. On average, 90% of participants had taken out a loan in the 30 days prior to baseline, with this being slightly more common in Pirimiti and Matawale than Likangala. Almost all participants had experienced one or more economic shock in the six months prior to baseline (median of three economic shocks per participant), indicating a high level of economic vulnerability in this population.

In terms of their PMTCT experience, most had accessed PMTCT services for a previous pregnancy (88%), with women in Likangala accessing PMTCT previously more often than women in the other two sites. On average, 88% of participants had disclosed their status to their partner and 79% had disclosed to their family and friends.

### Financial diary data

#### Inflows

There were 68,097 total cash transactions over 26 weeks. Roughly 10% of total transactions were inflows (Table 4). Earned cash income, or instances where women received money through service or labor, comprised 40.8% of total inflows with a median cash value of MWK 2,545 (USD 3.54). Yet only 13.7% of total inflows were reported as business-related. This highlights findings from the qualitative data that women are largely engaged in “piece work,” or informal and sporadic labor, to earn money. Particularly in Pirimiti and Likangala, participants did not consider engaging in piece work to be a form of business because of its varied and *ad hoc* nature.

**Table 4:** Overview of cash inflows and outflows by transaction type.

	Inflows			Outflows		
	Total Number	Median Value (MWK)	Median Value (USD)	Total Number	Median Value (MWK)	Median Value (USD)
Cash (Payment/Income)	2,766	2,545	3.54	60,363	303	0.42
Gift	1,541	2,034	2.83	270	407	0.57
Intra-household transfer	1,755	3,055	4.25	69	252	0.35
Debt repayment	26	991	1.38	268	1,524	2.12
Loan	469	2,034	2.83	89	1,021	1.42
Savings	177	3,055	4.25	251	1,021	1.42
Remittances	48	4,068	5.66	5	2,034	2.83

IHTs accounted for 25.9% of inflows with a median cash value of MWK 3,055 (USD 4.25). Cash gifts from non-household members represented an additional 22.7% of inflows. Taken together, cash from others not expected to be repaid accounted for nearly half of inflows (48.6%) and a greater portion of inflows than earned income alone.

These findings are strongly supported by the qualitative IDI data, which explored the crucial role of social support in participants’ lives. Notably, many participants described financial support from partners, family, and friends, which often facilitated their PMTCT R&A. Partners were more often described as providing financial support than family and friends, such as in this example provided by a participant from Likangala:

*“…my husband went out to fetch for piece work in town*. *When he was there he sent me 25*,*000 MWK*, *so that money is that which I used in those weeks*, *including transport for clinical trips*.*”*

Some participants sought out piece work only out of necessity, as illustrated by a participant from Pirimiti:

*“We suffer a lot when my husband gets sick because he is our main source of income*. *I do piece work when such scenarios happen to find money to get medication for him*.*”*

**Fig 2:**
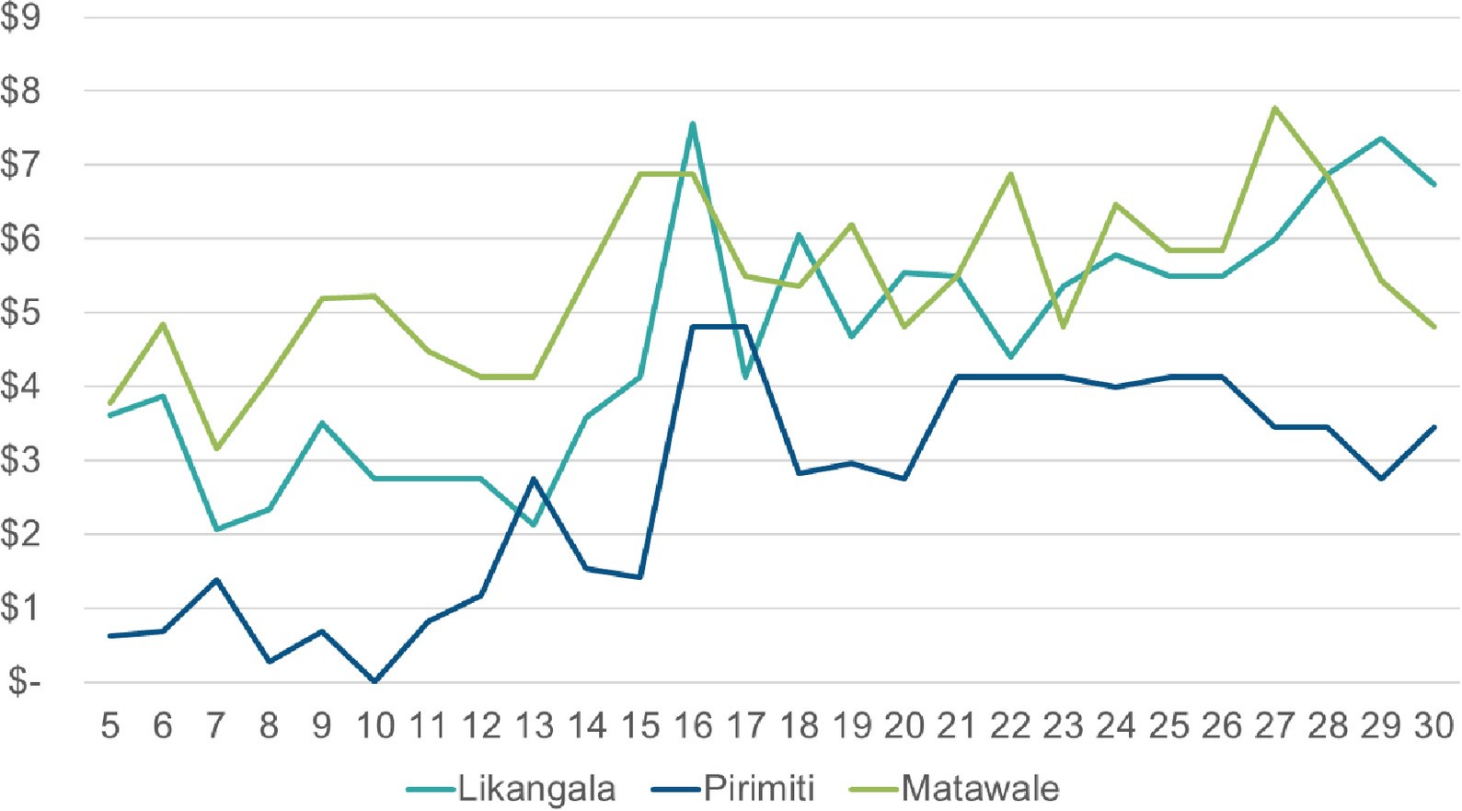
Median weekly cash income by site.

Over the 26 weeks included in this analysis, there were no notable differences in median weekly income across sub-groups. By site, Pirimiti consistently experienced the lowest median weekly cash income, though all sites experienced a slightly increasing slope over time (Fig 2). In particular, inflows began to increase after week 15 of the study, which coincided with the beginning of Malawi’s rainy season in November when agricultural piece work increased, especially in peri-urban and rural areas. As a participant from Matawale explained:

*“The amount is higher because this is the season for farming*, *so plenty of piece works are available*. *We are working in many farms almost every day*, *so you can see yourself that money is not a big problem now*.*”*

#### Outflows

Compared with inflows, cash outflows accounted for 90% of total cash transactions. In terms of transaction categories, instances where women purchased goods or services with cash represented 88.6% of outflows. Yet, the median cash value for cash outflows transactions was relatively small at MWK 303 (USD 0.42) compared to the median value of cash inflows. When participants spent cash, most transactions were for purchasing food/drink items (82.2%) such as fish or shellfish, and household items (12.6%) such as bath soap and matches (Table 5). Less than 2% of outflows were considered child expenses (e.g. children’s clothing/uniforms and school fees).

**Table 5:** Breakdown of outflows by category.

Types of Items Bought with Cash	Total # of Transactions	Median Value (MWK)	Median Value (USD)	Example Item Bought
Food or Drink	49,616	296	0.41	Fish/Shellfish
Household	7,602	296	0.41	Bath Soap
Child Expenses	1,031	496	0.69	Clothing
Transportation	824	991	1.38	Bicycle
Communication	442	200	0.28	Airtime
Medical/Health	319	200	0.28	Medication from Pharmacy
Agriculture	318	1,982	2.76	Field Labor
Discretionary	189	200	0.28	Offering at Church or Mosque

**Fig 3:**
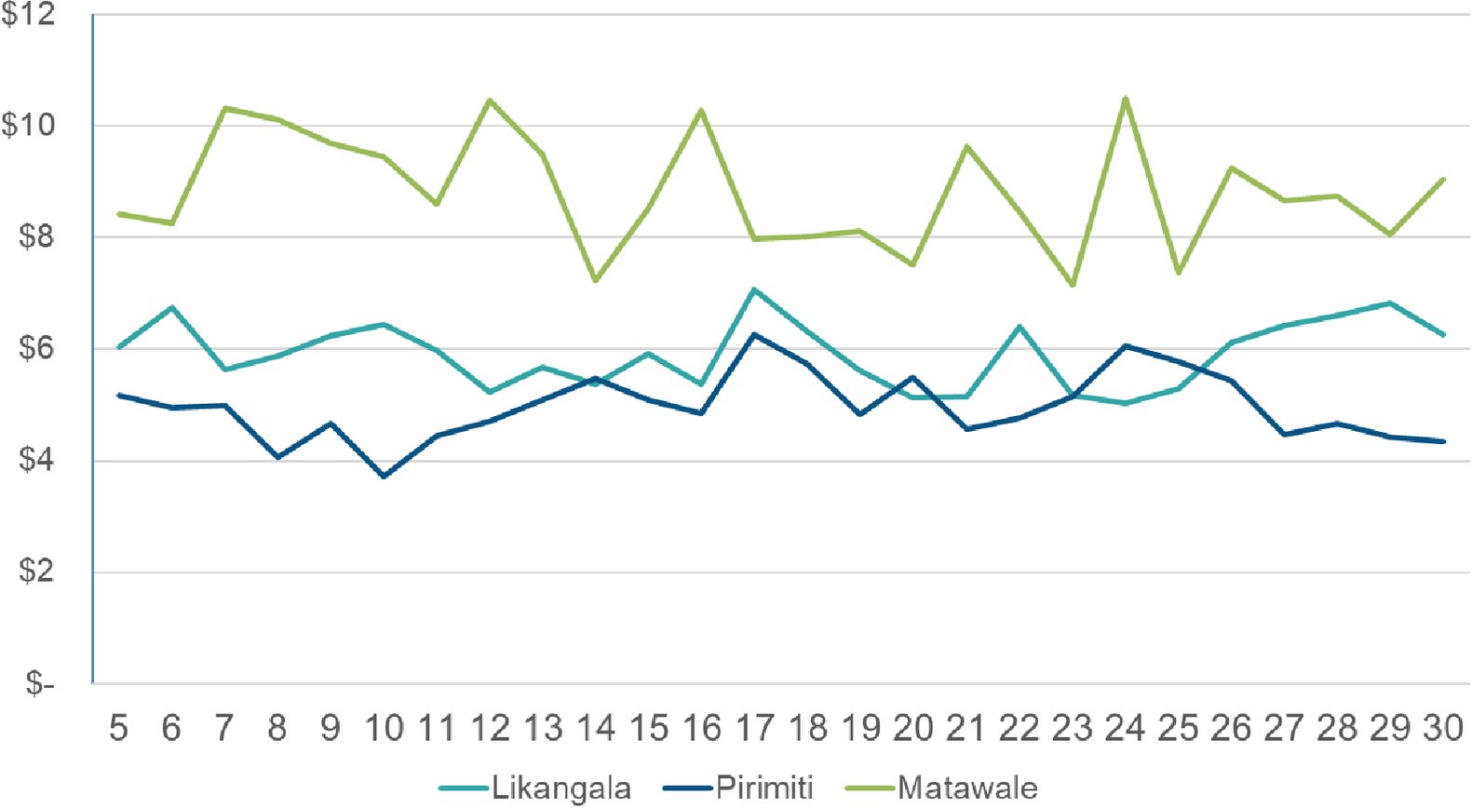
Median weekly cash outflows by site.

Median weekly cash outflows remained relatively consistent over time (Fig 3). Expenditures in Matawale, the urban site, were consistently higher than in the peri-urban and rural areas. There were no notable differences in outflows by sub-group.

Of all transactions, 7.0% were considered related to PMTCT, while an additional 28.7% were related to health more generally. Taken together, health-related expenditures accounted for more than one-third of all transactions. Outflows (7.2%) were more often considered PMTCT-related than inflows (5.7%). Looking specifically at spending on PMTCT, again, 88.8% of transactions were on food/drink, and transportation (5.1%) accounted for the second greatest portion of PMTCT-related transactions (Table 6).

**Table 6:** Breakdown of outflows for PMTCT by category.

Type of Expense	Total # of Transactions	Median Value (MWK)	Median Value (USD)	Example Item Bought
Food or Drink	3,879	296	0.41	Fish/Shellfish
Transportation	224	991	1.38	Bicycle
Household	156	200	0.28	Matches
Child Expenses	56	296	0.41	Formula
Medical/Health	55	296	0.41	Medication from Pharmacy

Qualitatively, participants described how they determined what constituted a PMTCT-related expense. These centered around money as a facilitator of PMTCT R&A, particularly in terms of money for food and transportation. For PWNM, it was important to have adequate food for two reasons. First, participants described how taking medication without food could lead to side effects, including dizziness and nausea, and any negative side effects could leave her without energy to take care of herself and her family. A participant in Pirimiti described the linkage between money, food, and R&A:

*“Because for me to stay in PMTCT care*, *I need money*. *I cannot take medication without eating any food*. *I need to make money in order for me to buy food and*, *therefore*, *be able to take medication*.*”*

The critical role of food was further emphasized by a participant in Likangala:

*“It is hard to take the medicine without eating first*. *You experience dizziness in the middle of the night if you took your medicine before eating first*. *These drugs are too strong to take without eating first*.*”*

Additionally, the emphasis on food/drink highlights counseling PWNM receive at health facilities on the importance of diverse and protein rich diets. A participant from Likangala explained how she put PMTCT messaging into practice:

*“…at the clinic they advise us to eat food of different groups*, *so we buy vegetables*, *tomato*. *If we have a lot of money*, *we buy meat*, *groundnut flour*. *These help us because our bodies stay healthy*. *Apart from that we also buy fish*, *beans*, *foods of different groups because that also helps us to stay healthy*.*”*

### PMTCT R&A

We measured adherence over the PMTCT cascade as PDC and found that participants across sites had an average 90% of PDC. This was slightly higher in Likangala (92%) than the other sites (89%, respectively). Participants enrolling in the study in the 2 months post-partum group had an average 84% of PDC compared with the other groups, which were 90% or higher. PDC was highest for participants enrolling in the 14 months post-partum group.

**Fig 4:**
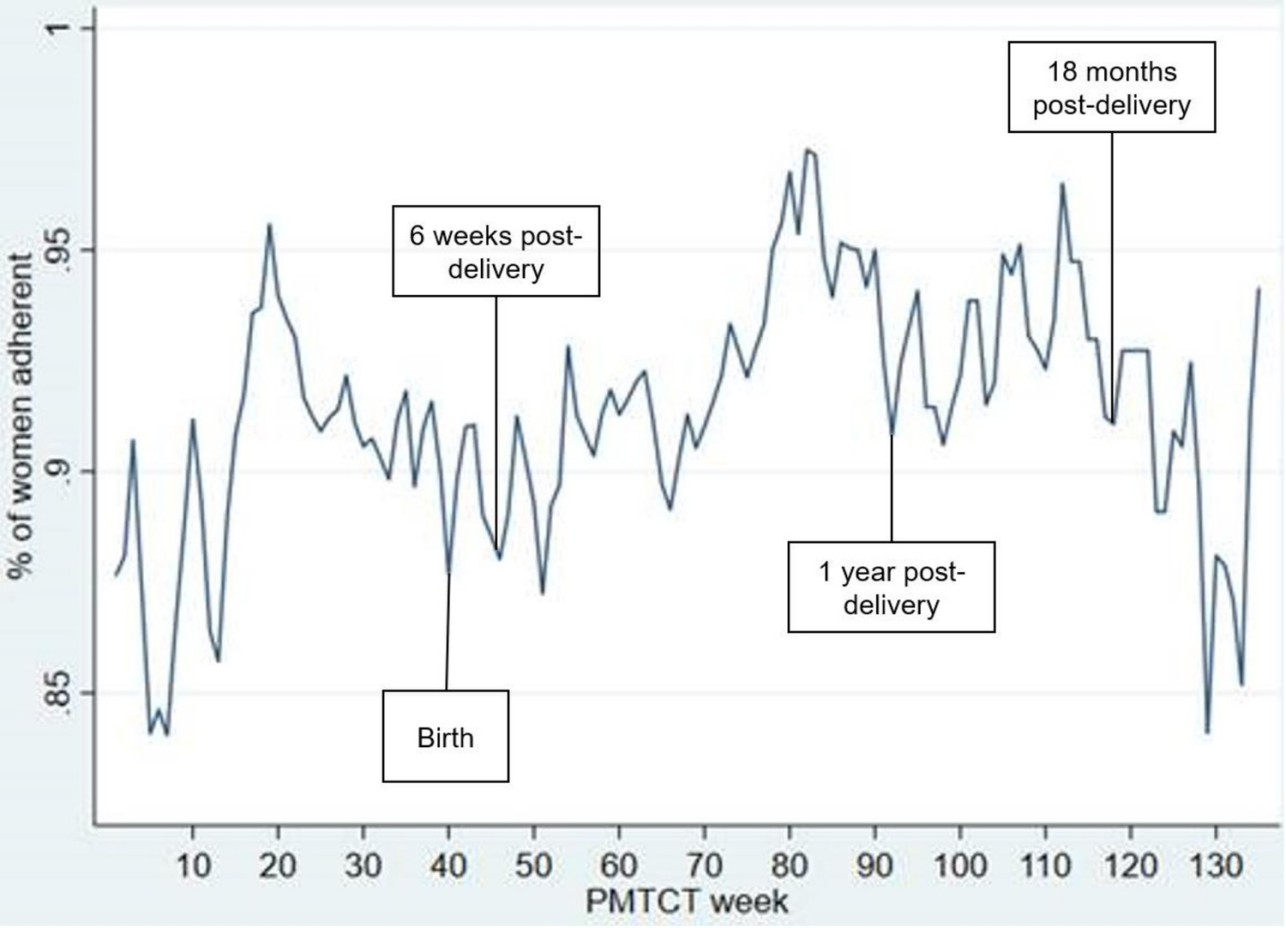
Proportion of women who were adherent over the PMTCT cascade.

Using the weekly binary adherence measure, we charted the proportion of women who were adherent each week across weeks in the PMTCT cascade for which sufficient data were available. This was to provide a sense for where along the cascade women are most vulnerable to non-adherence. We found that the proportion of women who were adherent fluctuated between 84% at its lowest early during pregnancy, and 97% at its highest around week 82. There were notable fluctuations in adherence during birth and approximately 12 weeks post-partum (week 52), which may implicate challenges in attending the child’s 6-week post-natal visit. After week 52, the proportion of adherent women slowly climbed to its pinnacle shortly before the child’s first birthday, where it stays above 90% for more than the next six months (Fig 4).

#### Survival analysis

**Fig 5:**
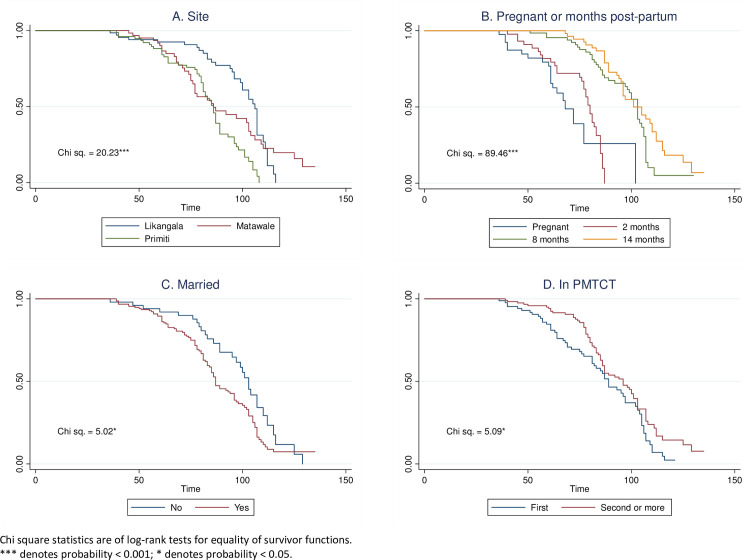
A–D. Kaplan-Meier curves representing time to first non-adherence by (A) site, (B) group, (C) marital status, and (D) prior experience with PMTCT.

Using the binary weekly adherence measure, we also examined time to first non-adherence across the PMTCT cascade. The Kaplan-Meier curves represent the estimated probabilities of being adherent up to a certain time point or beyond. They start at conception and cover pregnancy through approximately two years post-partum. The survival analyses represented in Fig 5A–5D indicate the first week in which participants missed four or more pills in a row. This gives a sense for the first time point along the PMTCT cascade when picking up pills becomes a challenge.

Each Kaplan-Meier plot (5a-d) includes a chi-square statistic of the log-rank test, indicating a difference between all curves on the plot. The larger the chi-square statistics, the greater the distance between the curves. For example, in Fig 5A, there was a statistically significant difference in time to first non-adherence by site (chi-square = 20.23, p<0.001). Although participants in Likangala were more likely to experience non-adherence before those in Matawale and Pirimiti, participants in Likangala had the highest probability of being adherent between approximately weeks 60 and 110 (approximately five to 16 months post-partum). There was also a statistically significant difference between the curves for time to non-adherence for participant groups in Fig 5B (chi-square = 89.46, p<0.001). Participants in the 14-month post-partum sub-group did not experience non-adherence until about week 70 (approximately seven months post-partum), compared with the pregnant and 2-month post-partum sub-groups experiencing first non-adherence at about weeks 40 and 45, respectively, which is around birth or the early post-partum period. In general, women enrolled at 8- or 14-months post-partum had a longer time to first non-adherence than those that enrolled during pregnancy or in the 2-month post-partum group.

The Kaplan-Meier curve in Fig 5C shows a statistically significant difference in the time to first non-adherence by marital status (chi-square = 5.02, p<0.05). Unmarried women had a higher likelihood of non-adherence earlier in the PMTCT cascade but had a higher probability of being adherent relative to married women further into the PMTCT cascade. Finally, there was a statistically significant difference in the Kaplan-Meier curves for women with prior experience with PMTCT services in Fig 5D (chi-square = 509, p<0.05). Women who had been in PMTCT in a previous pregnancy generally had a higher probability of being adherent further into the PMTCT cascade than women who were in PMTCT for their first time (i.e. new mothers or those previously not using PMTCT services).

In addition to using the Kaplan-Meier curves for exploring the time to non-adherence along the weeks of the PMTCT cascade for different participant groups, we fit a Cox proportional hazards regression model on time to first non-adherence controlling for the nine key covariates outlined in Table 7. From this analysis we found significant relationships between first non-adherence and site, sub-group, number of dependents, and cash inflows and outflows.

**Table 7:** Cox proportional hazard model.

	Hazard Ratio	SE	z	p>z
Age	0.9939	0.020	-0.3	0.767
Married	1.1713	0.272	0.68	0.496
Site				
Matawale	1.8401	0.534	2.1	0.036
Pirimiti	3.3431	0.875	4.61	**0.000**
Group				
2 months post-partum	0.8402	0.273	-0.54	0.592
8 months post-partum	0.1534	0.054	-5.37	**0.000**
14 months post-partum	0.0794	0.028	-6.91	**0.000**
Education				
Primary school	0.9159	0.272	-0.3	0.767
More than primary	1.2023	0.442	0.5	0.616
Years on ART	0.9939	0.040	-2.37	**0.018**
Number of direct dependents	0.8056	0.064	-2.74	**0.006**
Average Cash In	1.0001	0.000	2.25	**0.025**
Average Cash Out	0.9999	0.000	-2.17	**0.03**

*Significant effects in bold

Participants in Matawale and Pirimiti had 1.84 (*p* = 0.036) and 3.34 (*p*<0.00*)* times the hazard of non-adherence, respectively, over the PMTCT cascade than those in Likangala. Also, the hazard of non-adherence for participants in the 8- and 14-month post-partum groups were 15% (*p*<0.00) and 8% (*p*<0.00) of the hazard for those pregnant at baseline. We also found that each additional dependent decreased the hazard of non-adherence by an estimated 19.4% (*p* = 0.006) and that each additional year on ART decreased the hazard of non-adherence by 0.6% (*p* = 0.018).

Cash inflows and outflows were also statistically significantly associated with non-adherence over the PMTCT cascade after controlling for other covariates, but the hazard ratio estimates indicated a small, almost negligible practical association. However, in examining the qualitative data, we found some indication that higher inflows could be linked to non-adherence. For example, participants work to find piece work on or about the day of their appointment to have transportation fare or to be able to buy food to give them energy for a long walk to the facility. In some cases, this caused participants to postpone or miss their appointments, as a participant from Matawale explained:

*“Because of distance it is not possible to go to the hospital and come back in good time to do some work at home*, *especially when I have found a piece of work on the same day of appointment*. *As a result*, *I choose to work first and find money*, *then go to the hospital the following day*.”

By contrast, each additional 1,000 MWK spent per week decreased the hazard of non-adherence but only by <0.1% (*p* = 0.03).

#### Inflows and outflows by adherence status

**Fig 6:**
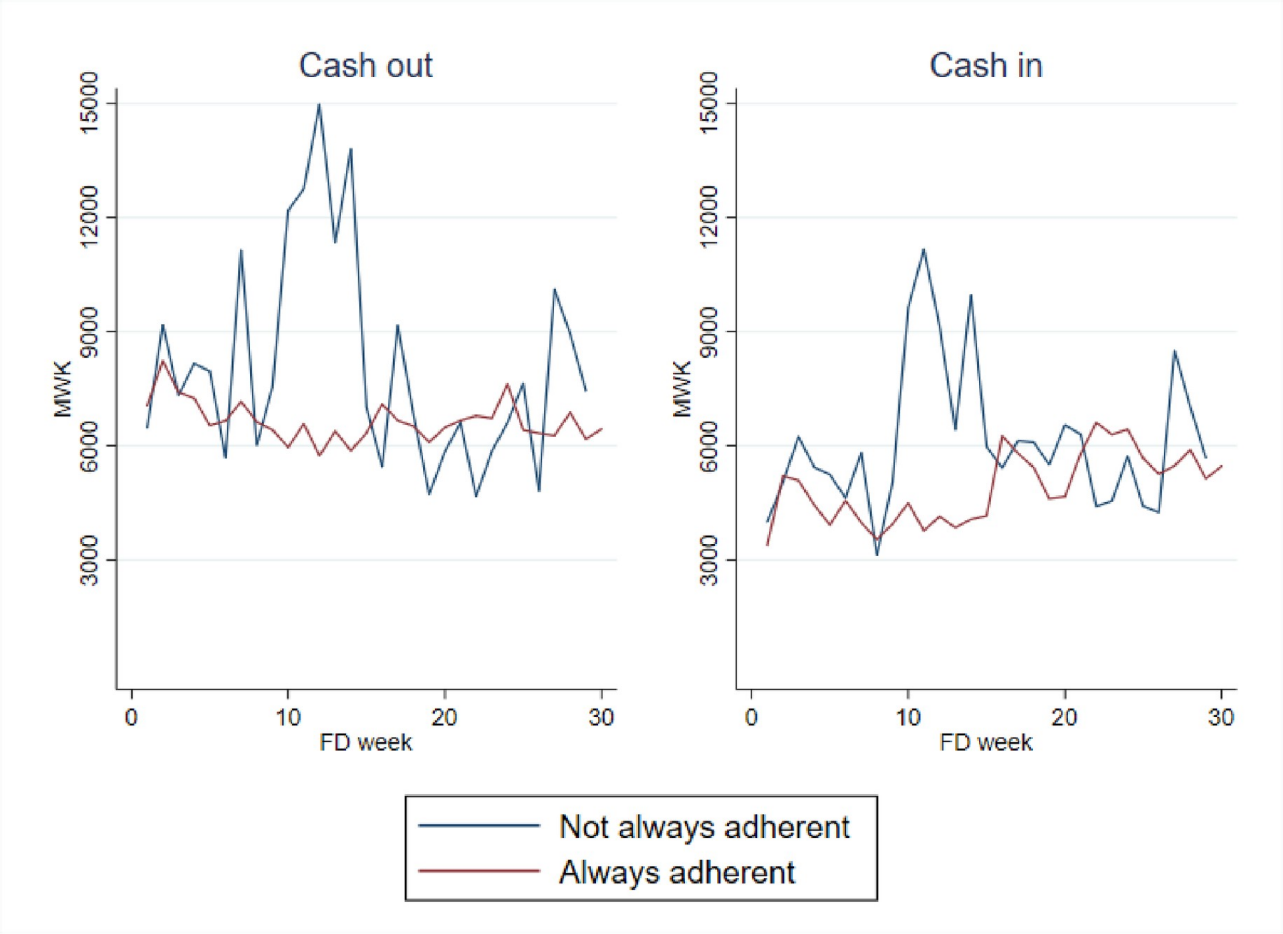
Cash outflows and inflows by adherence across the study window.

In an effort to better understand this relationship, we mapped cash inflows and outflows for participants who were always adherent compared with those who were not always adherent across the 26-week FD study window (Fig 6). To understand the relationship between these variables, we used a simple model regressing cash inflows and cash outflows separately on the binary adherence variable, controlling for study week. We included robust standard errors to adjust for autocorrelation. There were statistically significant differences in that those who were always adherent experienced relatively stable inflows (p = 0.002) and outflows (p = 0.006). Those who were not always adherent, on the other hand, had much greater variability in both their inflows and outflows.

The significance of the influence of cash inflows and outflows on PMTCT R&A is echoed by the qualitative data, which emphasizes one clear message: *money matters*. As a participant in Matawale explained:

*“I can’t lie*, *when money is available I become happy because it gives me the ability to buy my heart’s desires*. *I go to the clinic knowing in mind that there is food at home*, *and also the transport fee is in my pocket*.*”*

## Discussion

In many ways, the FD data validate what had previously only been anecdotal experiences for PWNM in Zomba. We found that participants at study sites are indeed a cash-poor population where resources are focused almost exclusively on meeting their family’s basic needs. Outflows consisted of small, near constant transactions, primarily for food/drink, while inflows were larger but reported at unpredictable intervals. Earning money through service or labor was the single largest source of inflows; however, the combined importance of money from IHTs and gifts highlights participants’ dependence on others for financial support that influences their PMTCT R&A.

Participants considered relatively few transactions to be related to their PMTCT care, the majority of which were outflows for food/drink. This highlights the importance of food for R&A, indicating that many participants take seriously the messaging from counselors on the importance of a diverse and protein-rich diet. The abundance of PMTCT-related outflows for fish/shellfish and groundnut flour––protein-rich foods that are considerably more affordable than meat and eggs in these communities––shows how women aimed to adhere to dietary guidance despite struggling to meet basic needs. The second most common PMTCT-related expenditure was transportation; however, we recognize that FD data does not accurately capture an estimate for transportation since lost opportunity costs are not considered. A significant portion of women in PMTCT walk to their appointments, regardless of the distance. In locations like Pirimiti and Likangala, this can take up to five hours each direction, or ten hours per day, which impinges substantially on livelihood commitments and the ability to find money or food for that day.

Qualitatively, participants communicated a determined commitment to their adherence, yet average PDC across sites and sub-groups remained moderate and below a clinically significant threshold of 95% or greater. Certain variables were related to non-adherence, including site and sub-group. Participants in Pirimiti may have been at greater risk of non-adherence since this was a private health facility and, for those who did not reside in the catchment area, some services were not provided free-of-charge. We also found that women who had been in PMTCT before had better adherence than first time PMTCT mothers, indicating that women returning to “the program,” as it is often called, may be more committed to their adherence. This could be a result of experiencing the benefits of PMTCT for another child, receiving consistent medical advice for a longer time-period, or it could be associated with having a more established routine around managing appointments and taking medication. The importance of having a routine may also be highlighted in similar findings from Malawi that Option B+ patients who started ART while pregnant were five times more likely to not return to the clinic than patients who started ART for their own health prior to the pregnancy [16]. These qualities have inspired the use of “expert mothers”, or older mothers with PMTCT experience, in Malawi, Nigeria, South Africa, and Zimbabwe to mentor younger and treatment naïve mothers to improve retention in PMTCT [17, 18].

Similarly, there were some differences in the proportion of women who were adherent across the PMTCT cascade. There was a notable drop in adherence after the child’s 6-week appointment that stayed low through at least four months post-partum. This could be linked to the general challenges of having a new baby. For some women, it may also be linked to non-disclosure [19, 20]. If family and friends are visiting the new child but the mother has not disclosed her status to her network, she may be less likely to adhere to her medication or pick-up schedule. Another consideration is that many women in this study found out about their status during their pregnancy and continued to struggle with their new reality as HIV-positive. A meta-analysis of retention issues in PMTCT reported that women initiating ART during pregnancy were at higher risk for LTFU [21]. Some women may decide to continue in care until the child’s six-week check-up when infant prophylaxis is discontinued and the child is usually tested for HIV, but then feel a lessened sense of importance in terms of their own adherence once she has cleared initial post-natal milestones.

Decreased cash outflows were also significantly associated with very slight increases in non-adherence. This is aligned with the idea that, as economic stability increases, both income and expenditures increase; as economic stability increases, we, in turn, expected economic barriers to R&A to reduce and adherence to improve. In addition, given that the majority of expenditure was on food, which participants frequently noted as being positively linked with adherence, it seems reasonable that increased expenditure could improve adherence. Interestingly, higher cash inflows were associated with greater non-adherence, which was unexpected. Our qualitative findings, however, indicate that earning money may reduce adherence if it occurs when participants should be at the facility. Participants’ reliance on others for financial support also likely indicates that they need financial and material support from partners and others to overcome the easily and consistently articulated economic barriers that influence their care-seeking behaviors. Despite that ART and counselling are free in Malawi, it is clear that PWNM struggle with the basics of being able to afford transportation to appointments and food with which to take medication.

ES interventions can fill this space by providing an additional avenue for support, and participants were enthusiastic about potential interventions that would mitigate their barriers to care. Food support interventions have been effective at improving retention and adherence among general HIV-positive populations [22] and would also be well-positioned to address the barriers described by women in our sample. Compared to food support needs among the general population, the provision of such support may be more feasible for the distinct PMTCT time window. However, a direct food transfer is just one option for delivering this support. Participation in savings groups, where small groups of individuals collectively save and use pooled savings to make low interest loans to group members, could serve as a source of cash. This may translate into a greater ability to pay for PMTCT-related costs, including food, through loans or after a share-out. Moreover, savings groups typically include social funds to assist members in times of need, which could be useful to pay for emergency transport to facilities during labor. Savings groups may also expose PWNM to empowering training, resources, and social support that could have a longer-term influence on their lives.

Financial consistency and predictability also have some bearing on adherence; therefore, implementing interventions like income generating activities that can smooth consumption and savings groups to support business endeavors may have lasting effects on PMTCT outcomes. These interventions, however, require participants to invest significant time before benefits are achieved, which may make them impractical for PMTCT adherence specifically. However, the need for PWNM to be on ART for life, coupled with high parity in this population makes them reasonable interventions in support of PMTCT objectives.

### Limitations

This study has several important limitations. First, the study was exploratory in nature and was not designed with the specific intention of quantitatively assessing the correlations provided in this paper. The relatively small sample size may be contributing to non-significant or underpowered results. Second, the FD data was collected weekly and could have been subject to recall bias whereby some transactions may have been forgotten or misremembered. Third, much of the study data, including FD transaction data, was based on self-report, which could have resulted in over- or under-reporting of some transactions. Finally, an important factor that was not taken into consideration in this analysis is how women’s preference for health care utilization may influence their spending. However, the authors found in separate qualitative analyses (not presented here) that most women did not question their need for PMTCT services once enrolled. Even when women had concerns about continuing with PMTCT care due to economic factors, they still continued with the program given an overwhelming desire to protect their health as well as that of the child’s. This, combined with the regimented nature of PMTCT programs, likely attenuated any influence of personal preference for health care utilization on spending.

## Conclusions

PWNM in this context control very small amounts of cash. Income is erratic and expenses are focused on meeting basic household needs, especially food. PMTCT expenses comprise a small proportion of overall expenses and are focused on obtaining nutritious food and, to a lesser extent, transportation to the health facility. As such, higher overall spending is significantly associated with greater adherence, though the magnitude of association found in this study was minor. Economic stability is also associated with greater adherence, and women in this population would benefit from ES support to bolster and stabilize their economic lives and ultimately improve their R&A. Average adherence varies over the PMTCT cascade, which could reflect financial as well as social and practical influences on PMTCT decision-making which warrant further exploration.

## Supporting information

S1 File Baseline questionnaire in English. (PDF)

S2 File Baseline questionnaire in Chichewa. (PDF)

S3 File Financial diary debriefing form in English. (PDF)

S4 File Financial diary debriefing form in Chichewa. (PDF)

S5 File IDI guide in English. (PDF)

S6 File IDI guide in Chichewa. (PDF)
