# Supplementary material for: Using financial diaries to understand the economic lives of HIV-positive pregnant women and new mothers in PMTCT in Zomba, Malawi
Source: PLoS One. 2021 Jul 30;16(7):e0252083. doi: 10.1371/journal.pone.0252083 (PMC8323884; doi:10.1371/journal.pone.0252083)
Supplement: S1 File — (PDF) [file pone.0252083.s001.pdf]

FHI 360

General Questionnaire for Financial Diaries Activity
Version 2.0; May 29, 2018

Title:	*Financial Diaries to Understand the Economic Context of PMTCT Retention and Adherence in Malawi*
Sponsor:	*FHI 360 and USAID*
Address:	*FHI 360, 359 Blackwell St, Suite 200, Durham, NC 27701 USA*

Notes for researchers:

No.	Question	Response	Code
Int	Interviewer ID	[fill-in]	
Today	Date of interview	*[calendar automated to mark system date, but can be modified by data collector]*	
Site	Site/Location	0 = Likangala Health Centre (Rural)<br>1 = Pirimiti Community Hospital (Peri-urban)<br>2 = Matawale Health Centre (Urban)	
PID1	Participant ID	[fill-in]<br><br>*Note to programmer:*<br>*1001-1200 only valid if site=0*<br>*2001-2200 only valid if site=1*<br>*3001-3200 only valid if site=2*	

SECTION 1: Respondent information

I would like to begin by collecting some background information from you.

No.	Question	Response	Code
101.	How old are you?	[fill-in, integer only]	constraint 18-49
102.	What is your marital status?	1 = Married (monogamous)<br>2 = Married (polygamous)<br>3 = Single<br>4 = Divorced<br>5 = Separated<br>6 = Widowed<br>7 = Domestic partner<br>99 = Other __________________	
103.	Have you ever been to school?	0 = No<br>1 = Yes	
104.	What is the highest grade you completed?	1 = Primary school<br>2 = Lower secondary<br>3 = Upper secondary<br>4 = Trade school<br>5 = University or higher<br>99 = Other __________________	if Q104=1
105.	Are you able to read?	1 = Cannot read at all<br>2 = Able to read part of the sentence	

	Interviewer shows woman card with sentence to read aloud.<br><br>*I buy maize from a vendor in the market.*	3 = Able to read the entire sentence<br>4 = Blind/visually impaired	
106.	What is your religion?	1 = Christian<br>2 = Muslim<br>3 = Traditional<br>4 = No religion<br>99 = Other _______________	
107.	What is your ethnic group?	1 = Chewa<br>2 = Lomwe<br>3 = Yao<br>4 = Ngoni<br>5 = Tumbuka<br>6 = Sena<br>7 = Tonga<br>8 = Nyanja<br>99 = Other _______________	

SECTION 2: Household Characteristics

Now, let's talk about your household.

No.	Question	Response	Code
201.	I would like to record the number of people who live in this household. A household is a group of people who live together, pool their money, and eat at least one meal together each day.<br><br>With that in mind, how many members does the household have?	A. Seven or more<br>B. Six<br>C. Five<br>D. Four<br>E. One, two, or three	
201a	Is the head of household a man or a woman?	0 = Man<br>1 = Woman	
202.	Is the (oldest) female head/spouse able to read and write in Chichewa or English?<br><br>*Program as a hint*: While most homes have one female head of household, some have more than one. If that is the case, we are asking about the **oldest female head of household.** The oldest female head of household may not be the oldest female in the house (ex. Grandmother may be living there but is not the head of household).	A. No<br>B. Yes, only Chichewa<br>C. Yes, English (regardless of Chichewa)<br>D. No female head/spouse	
203.	The floor of the main dwelling is predominantly made of what material?	A. Smoothed mud or sand<br>B. Smooth cement, wood, tile or other	
204.	The outer walls of the main dwelling of the household are predominantly made of what material?	A. Mud (yomata) or grass<br>B. Mud brick (unfired)<br>C. Compacted earth (yamdindo), burnt bricks, concrete, wood, iron sheets, or other	
205.	The roof of the main dwelling is predominantly made of what material?	A. Grass, plastic sheeting, or other<br>B. Iron sheets, clay tiles, or concrete	

206.	What kind of toilet facility does the household use?	A. None, traditional latrine without roof shared with other households, or other<br>B. Traditional latrine without roof only for household members<br>C. Traditional latrine with roof shared with other households<br>D. Traditional latrine with roof only for household members, VIP latrine, or flush toilet	
207.	What is the household's main source of lighting fuel?	A. Collected firewood, purchased firewood, grass, or gas<br>B. Paraffin, or other<br>C. Battery/dry cell (torch), candles, or electricity	
208.	Do any members of the household sleep under a mosquito net to protect against mosquitos at some time during the year?	A. No<br>B. Yes	
209.	Does the household own any tables?	A. No<br>B. Yes	
210.	Does the household own any beds?	A. No<br>B. Yes	
211.	How many children under 18 live in this household?	___ ___ children under 18	constraint: 0-20
212.	Of those, how many are your direct dependents?<br><br>By direct dependents, we mean children that you are legally or financially responsible for taking care of everyday.	___ ___ children	constraint: 212=<211

SECTION 3: Income Sources & Financial Management

For these next questions, I would like to speak with you about the money and resources in your household, especially the money and resources that you personally control.

No.	Question	Response	Code
301.	Do you personally own any land?	0 = No<br>1 = Yes, joint ownership<br>2 = Yes, sole ownership	
302.	Do you personally own any animals?	0 = No<br>1 = Yes, joint ownership<br>2 = Yes, sole ownership	
303.	Do you personally own a mobile phone?	0 = No<br>1 = Yes, joint ownership<br>2 = Yes, sole ownership	
304.	Do you personally own any house?	0 = No<br>1 = Yes, joint ownership<br>2 = Yes, sole ownership	
304a	Do you personally rent any land or buildings?	0 = No<br>1 = Yes, joint rental<br>2 = Yes, I rent alone	

304b	Do you personally have a bank account at a formal bank?	0 = No<br>1 = Yes<br>88 = DK/Refuse	
305.	Have you worked to earn money in the past 30 days?	0 = No → **Skip to 310**<br>1 = Yes	
306.	What occupation were you involved in to earn money in the past 30 days?<br><br>**Select the best option for each different occupation.**	1 = Agriculture<br>2 = Fishing<br>3 = Health Worker<br>4 = Skilled or unskilled manual labor<br>5 = Domestic service<br>6 = Educator<br>7 = Trader/Seller<br>8 = Professional/technical/managerial<br>99 = Other _______________	
307.	How would you characterize your main occupation in the past 30 days?	1 = Permanent job<br>2 = Temporary job<br>3 = Seasonal job<br>4 = Daily wage<br>5 = Self-employed<br>99 = Other _______________	
308.	What was your estimated income in the past 30 days in kwacha?<br><br>If the participant doesn't know, enter 88.	[fill in number]	
309.	How regularly do you earn money?	1 = Daily<br>2 = Weekly<br>3 = Monthly<br>4 = Seasonally<br>5 = Unpredictable intervals<br>99 = Other _______________	
310.	If you have not worked in the past 30 days, have you worked at any time during the past year?	0 = No → **Skip to 312**<br>1 = Yes	relevance: Q305 is NOT 1
311.	What occupation were you involved in to earn money in the past year?<br><br>**Select the best option for each different occupation.**	1 = Agriculture<br>2 = Fishing<br>3 = Health Worker<br>4 = Skilled or unskilled manual labor<br>5 = Domestic service<br>6 = Educator<br>7 = Trader/Seller<br>8 = Professional/technical/managerial<br>99 = Other _______________	relevance: Q305 is NOT 1
311a	Including yourself, how many people in your household make money that goes toward household expenses?	[integer fill-in]	
312.	Do you have a husband/partner?	0 = No → **Skip to 316**<br>1 = Yes	
312.3	312a. Does your husband or partner live with you?	0 = No<br>1 = Yes	relevance: if 312=1
313.	Has your husband/partner worked in the past 30 days?	0 = No → **Skip to 316**<br>1 = Yes<br>88 = DK/Refused → **Skip to 316**	

314.	What occupation was he involved in to earn money in the past 30 days?<br><br>**Select the best option for each different occupation.**	1 = Agriculture<br>2 = Fishing<br>3 = Health Worker<br>4 = Skilled or unskilled manual labor<br>5 = Domestic service<br>6 = Educator<br>7 = Trader/Seller<br>8 = Professional/technical/managerial<br>99 = Other ________________	
315.	How would you characterize his main occupation in the past 30 days?	1 = Permanent job<br>2 = Temporary job<br>3 = Seasonal job<br>4 = Daily wage<br>5 = Self-employed<br>88 = DK/Refused<br>99 = other ________________	
316.	Is there anyone else in your household that earns an income and contributes to household finances?<br><br>**If there are multiple other people in the household that earn an income and contributes to the household finances, pick the individual who contributes the most.**	0 = No → **Skip to 320**<br>1 = Yes	
317.	What is their relationship to you?	1 = Parent<br>2 = Child<br>3 = Sibling<br>4 = Uncle or Aunt<br>5 = Niece or Nephew<br>99 = other ________________	
318.	What occupation was s/he involved in to earn money in the past 30 days?<br><br>**Select the closest option for each different occupation.**	1 = Agriculture<br>2 = Fishing<br>3 = Service or salesperson<br>4 = Crafts or tradesperson<br>5 = Construction<br>6 = Health worker<br>7 = Educator<br>8 = Other professional<br>88 = DK/Refused<br>99 = Other ________________	
319.	How would you characterize his/her occupation in the past 30 days?	1 = Permanent job<br>2 = Temporary job<br>3 = Seasonal job<br>4 = Daily wage<br>5 = Self-employed<br>88 = DK/Refused<br>99 = Other ________________	
320.	Who manages the household finances?	1 = Respondent<br>2 = Husband/partner<br>3 = Manage finances jointly<br>99 = Other __________________	
321.	Did your household follow a budget in the past 30 days?	0 = No → **Skip to 324**<br>1 = Yes<br>88 = DK/Refused → **Skip to 324**	

322.	Did any expenses fall outside your budget in the past 30 days?	0 = No → **Skip to 324**<br>1 = Yes<br>88 = DK/Refused → **Skip to 324**	
323.	What were the expenses?<br><br>*Record the items/services, not a cash amount, that fell outside the budget in English.*	[text fill in]	
324.	Did you have a savings goal in the past 30 days?	0 = No → **Skip to 326**<br>1 = Yes	
325.	Did you meet your savings goal over the past 30 days?	0 = No<br>1 = Yes → **Skip to 327**	
326.	Did you save any money in the past 30 days?	0 = No<br>1 = Yes	
327.	What is the current total balance of your savings in kwacha, including money saved more than 30 days ago?<br><br>If the participant doesn't know, enter 88.	[fill-in number]	
328.	Do you have any outstanding loans?	0 = No → **Skip to Section 4**<br>1 = Yes<br>88 = DK/Refused	
329.	Did you take out any loans over the past 30 days?	0 = No → **Skip to 332**<br>1 = Yes	
330.	What is the total value of loans you took out over the past 30 days in kwacha?<br><br>If the participant doesn't know, enter 88.	[fill-in number]	
331.	Were the loans a part of your budget plan?	0 = No<br>1 = Yes	relevance: skip if Q321=0 or 88
332.	What is the current total balance of the loans you owe in kwacha, including loans taken out more than 30 days ago?<br><br>If the participant doesn't know, enter 88.	[fill-in number]	

SECTION 4: Economic Shocks and Benefits

For these next questions, I would like to discuss anything your household lost or gained.

No.	Question	Response	Code
401.	Let's first discuss any losses your household may have experienced. Think about the past 6 months. I am going to read a list of experiences and as I read them please let me know if they happened to your household in that time:<br><br>*Select all that apply.*		
	A. Costly medical treatment or funeral expenses for anyone in the household<br>B. Loss of income to the household because a member of the household became seriously ill, was injured, or died<br>C. A member of the household who normally contributes to finances lost their job<br>D. The business of anyone in the household failed<br>E. Any member of the household capable of working left the house because of marriage, divorce, or abandonment		

	F. There was destruction or loss of property due to theft, robbery, or fire<br>G. Crops belonging to the household failed<br>H. There were losses of any type due to natural disaster (crop failure, livestock death, other)<br>I. Household experienced loss due to deflation in the price of agricultural products<br>J. There was a loss or decrease in the amount of remittances<br>K. Urgent repair to your house or property<br>L. Other shocks [fill-in]<br>M. None	
402.	Now, I'd like to discuss anything your household may have gained. In the past 6 months, did any member of your household:<br><br>*Select all that apply.*	
	A. Get a new job<br>B. Start to receive money or grants as aid from the government or non-governmental agencies<br>C. Receive an inheritance, large gift, winnings, dowry, etc.<br>D. Receive a scholarship<br>E. Receive an increase in the amount of remittances<br>F. Experience an increase in the household's production or prices received for agricultural products<br>G. Other gains [fill in]<br>H. None	

SECTION 5: PMTCT Experience

Now I would like to speak with you about your experience accessing treatment and services to prevent mother-to-child transmission of HIV.

No.	Question	Response	Code
	**GENERAL**		
501.	Have you disclosed your HIV status to your husband/partner?	0 = No<br>1 = Yes<br>2 = No partner<br>88 = DK/Refused	
502.	Have you disclosed your HIV status to any other family or friends?	0 = No<br>1 = Yes<br>88 = DK/Refused	
503.	Are you currently pregnant?	0 = No → **Skip to 505**<br>1 = Yes	
504.	How many weeks pregnant are you?<br><br>If the participant doesn't know, enter 88.	[Fill in number] → **Skip to 506**	constraint: 1-45
505.	How long ago was your baby born?	1 = Days ago<br>7 = Weeks ago<br>30 = Months ago	if Q503=0
505a.	How many days/weeks/months ago was your baby born?	[fill in integer]	if Q503=0
	**Testing/Diagnosis**		
506.	Did you learn you were HIV+ before or during this/your most recent pregnancy?	1 = Before this pregnancy<br>2 = During this pregnancy	
507.	What year were you diagnosed with HIV?	[fill in integer]	constraint: 1985-2019 OR 8888

	Try and help the participant remember what month and year she was diagnosed with HIV. If she doesn't remember, have her make her best guess.<br>*If they don't know enter 8888*		
507a.	What month were you diagnosed with HIV?<br><br>*Try and help the participant remember what month and year she was diagnosed with HIV. If she doesn't remember, have her make her best guess.*	1 = January<br>2 = February<br>3 = March<br>4 = April<br>5 = May<br>6 = June<br>7 = July<br>8 = August<br>9 = September<br>10 = October<br>11 = November<br>12 = December<br>88 = Don't know	constraint: 1-12 or 88
508.	Were you tested for HIV as part of antenatal care for this/your most recent pregnancy?	0 = No<br>1 = Yes<br>88 = DK/Refused	relevance: ask if Q506=2
	**Prior PMTCT experience**		
509.	Is this your first pregnancy/child?	0 = No<br>1 = Yes → **Skip to 511**	
510.	Did you use PMTCT services for any pregnancies other than the current/most recent one?	0 = No<br>1 = Yes<br>88 = DK/Refused	relevance: ask if Q509=0 AND Q506=1
	**Maternal ART**		
511.	Have you ever taken ART?	0 = No → **Skip to 518**<br>1 = Yes	
512.	What year did you start taking ART?<br><br>*Try and help the participant remember what month and year she started taking ART. If she doesn't remember, have her make her best guess.*<br>*If they don't know enter 8888*	[fill in integer]	relevance: if Q511=1<br><br>Constraint: >=Q507 =<2019 OR 8888
512a.	What month did you start taking ART?<br><br>*Try and help the participant remember what month and year she started taking ART. If she doesn't remember, have her make her best guess.*<br>*If they don't know enter 88*	[fill in integer]	relevance: if Q511=1<br><br>constraint 1-12 OR 88
513.	Did you begin taking ART right away when you were diagnosed with HIV?	0 = No<br>1 = Yes	if Q511=1
514.	Have you stopped taking ART at any point during this pregnancy?	0 = No → **Skip to 516**<br>1 = Yes	Q503=1

514a.	Have you stopped taking ART at any point during your most recent pregnancy or while breastfeeding your baby?	0 = No **→ Skip to 516**<br>1 = Yes	Q503=0
515.	Why did you stop?<br><br>*Note to interviewer: allow respondent to answer freely, then select option that fits response*	1 = Low motivation<br>2 = Too busy, other priorities<br>3 = Did not feel well, side effects<br>4 = Felt healthy, treatment not necessary<br>5 = Not enough food to take with pills<br>6 = Transportation costs to pick-ups<br>7 = Distance to facility<br>8 = Lack of partner support<br>9 = Challenges with health facility services and/or staff<br>10 = Concerns about stigma<br>88 = DK/Refused<br>99 = other _______________	if Q514=1
516.	Are you currently taking ART?	0 = No<br>1 = Yes **→ Skip to 518**	
517.	Why not?<br><br>*Note to interviewer: allow respondent to answer freely, then select option that fits response*	1 = Low motivation<br>2 = Too busy, other priorities<br>3 = Did not feel well, side effects<br>4 = Felt healthy, treatment not necessary<br>5 = Not enough food to take with pills<br>6 = Transportation costs to pick-ups<br>7 = Distance to facility<br>8 = Lack of partner support<br>9 = Challenges with health facility services and/or staff<br>10 = Concerns about stigma<br>88 = DK/Refused<br>99 = other _______________	if Q516=0
518.	With this/your most recent pregnancy, have you missed any PMTCT-related appointments or medication pick-ups?	0 = No **→ Skip to 520 or 521 (see Q503)**<br>1 = Yes, missed appointments<br>2 = Yes, missed pick-ups<br>3 = Yes, missed both	
519.	What was the main reason(s) for the missed visits? (select all)<br><br>*Note to interviewer: allow respondent to answer freely, then select option that fits response*	1 = Low motivation<br>2 = Too busy, other priorities<br>3 = Did not feel well, side effects<br>4 = Felt healthy, treatment not necessary<br>5 = Not enough food to take with pills<br>6 = Transportation costs to pick-ups<br>7 = Distance to facility<br>8 = Lack of partner support<br>9 = Challenges with health facility services and/or staff<br>10 = Concerns about stigma	if Q518=1,2 OR 3

		88 = DK/Refused<br>99 = other _______________	
	**PREGNANT WOMEN ONLY (see Q503)**		
520.	Do you plan to breastfeed when the baby is born?	0 = No<br>1 = Yes<br>88 = DK/Refused<br>**For all responses, Skip to Section 6**	relevance: Q503=1
	**POSTPARTUM WOMEN ONLY (see Q503)**		
521.	How old is your baby now?	1 = Days old<br>7 = Weeks old<br>30 = Months old<br>999 = Baby did not survive	relevance Q503=0
521a.	How many days/weeks/months old is your baby?	[fill in integer]	if Q521=1, 7 or 30
522.	How old was the child when he/she died?	1 = Days old<br>7 = Weeks old<br>30 = Months old<br>999 = Child died at birth → **Skip to section 6 (endline ONLY)**	relevance Q521=999
522a	How many days/weeks/months old was your baby when he/she died?	[fill in integer]	Q522=1, 7 or 30
523.	Did your baby begin ART immediately after birth?	0 = No → **Skip to 526**<br>1 = Yes	relevance Q503=0<br><br>Q522!=999
524.	Did your baby continue ART for the full 6 weeks?	0 = No<br>1 = Yes → **Skip to 526**	relevance Q503=0<br><br>Q523=1<br><br>babyage >=42<br>OR<br>deathage >=42
525.	What was the main reason you stopped?<br><br>*Note to interviewer: allow respondent to answer freely, then select option that fits response*	1 = Low motivation<br>2 = Too busy, other priorities<br>3 = Baby did not feel well, side effects<br>4 = Felt healthy, treatment not necessary<br>5 = Not enough food to take with pills<br>6 = Transportation costs to pick-ups<br>7 = Distance to facility<br>8 = Lack of partner support<br>9 = Challenges with health facility services and/or staff<br>10 = Concerns about stigma<br>88 = DK/Refused<br>99 = other _______________	relevance Q503=0 AND Q524=0<br><br>Q522!=999

526.	Was your baby's HIV status tested at 6 weeks?	0 = No → **Skip to 528**<br>1 = Yes<br>88 = DK/Refused → **Skip to 528**	relevance Q503=0<br><br>babyage >=42<br><br>Q522!=999
527.	Are you willing to share the result of your baby's HIV test?	0 = Negative<br>1 = Positive<br>2 = Not willing to share<br>3 = Don't know<br>**Skip to 529 for all responses**	relevance Q503=0<br><br>Q526=1<br><br>Q522!=999
528.	Why was your child not tested at 6 weeks?<br><br>*Note to interviewer: allow respondent to answer freely, then select option that fits response*	1 = Low motivation<br>2 = Too busy, other priorities<br>3 = Baby did not feel well, side effects<br>4 = Baby seemed healthy, testing not necessary<br>5 = Transportation costs to clinic<br>6 = Distance to facility<br>7 = Lack of partner support<br>8 = Challenges with health facility services and/or staff<br>9 = Concerns about stigma<br>88 = DK/Refused<br>99 = other _______________	relevance Q503=0<br><br>Q526!=1<br><br>Q522!=999
529.	Are you currently breastfeeding?	0 = No<br>1 = Yes	relevance Q503=0<br><br>Q521!=999
530.	Are you feeding your baby any foods or liquids other than breastmilk?	0 = No → **Skip to 533**<br>1 = Yes	relevance Q503=0<br><br>Q521!=999
531.	What else are you feeding your baby?<br><br>Select all that apply.	1 = Plain water<br>2 = Juice or juice drinks<br>3 = Clear soup<br>4 = Milk such as tinned, powdered, or fresh animal milk<br>5 = Infant formula<br>6 = Yogurt<br>7 = Bread, rice, noodles, porridge or other foods made from grains<br>8 = Fruits<br>9 = Vegetables<br>10 = Animal meat<br>99 = Other ___________________	relevance Q530=1
532.	How old was your baby when you introduced these foods?	1 = Days old<br>7 = Weeks old<br>30 = Months old<br>88 = DK/Refused	relevance Q503=0<br><br>Q530=1

			Q521!=999
532a.	How many days/weeks/months old was your baby when you introduced these foods?	[fill in integer]	Q532=1, 7 or 30
533.	Did you received guidance from your doctors and nurses on how to breastfeed your baby to prevent mother-to-child-transmission?	0 = No<br>1 = Yes	relevance Q503=0 AND Q521!=999
534.	Did/do you have challenges breastfeeding your child?	0 = No **→ Skip to Section 6**<br>1 = Yes	relevance Q503=0 AND Q521!=999
535.	What were they?<br><br>*Select all that apply.*<br><br>*Note to interviewer: allow respondent to answer freely, then select options that fit the response.*	1 = Breast conditions (engorged breasts, cracked nipples, etc.)<br>2 = Not producing enough milk<br>3 = Fear of transmitting HIV to child<br>4 = Concerns about stigma<br>5 = Oral thrush in baby's mouth<br>6 = Lack of support from family<br>7 = Returned to work and could not sustain breastfeeding<br>88 = DK/Refused<br>99 = Other ___________________	relevance<br><br>Q534=1

SECTION 6: Household Food Security & Dietary Diversity

Now, I would like to talk about the food that is consumed by your household.

	Question	Response	Code
601.	In the past 30 days, was there ever no food to eat of any kind in your house because of lack of resources to get food?	0 = No **→ Skip to 603**<br>1 = Yes	
602.	How often did this happen in the past 30 days?	1 = Rarely (1 – 2 times)<br>2 = Sometimes (3 – 10 times)<br>3 = Often (more than 10 times)	
603.	In the past 30 days, did you or any household member go to sleep at night hungry because there was not enough food?	0 = No **→ Skip to 605**<br>1 = Yes	
604.	How often did this happen in the past 30 days?	1 = Rarely (1 – 2 times)<br>2 = Sometimes (3 – 10 times)<br>3 = Often (more than 10 times)	
605.	In the past 30 days, did you or any household member go a whole day and night without eating anything at all because there was not enough food?	0 = No **→ Skip to 607**<br>1 = Yes	
606.	How often did this happen in the past 30 days?	1 = Rarely (1 – 2 times)<br>2 = Sometimes (3 – 10 times)<br>3 = Often (more than 10 times)	
607.	Now I would like to ask you about the types of foods that you or anyone else in your household ate yesterday during the day and at night.<br><br>Select all that apply.<br><br>A. Any [INSERT ANY LOCAL FOODS, E.G. UGALI, NSHIMA], bread, rice noodles, biscuits, or any other foods made from		

	millet, sorghum, maize, rice, wheat, or [INSERT ANY OTHER LOCALLY AVAILABLE GRAIN]?<br><br>B. Any potatoes, yams, manioc, cassava, or any other foods made from roots or tubers?<br><br>C. Any vegetables?<br><br>D. Any fruits?<br><br>E. Any beef, pork, lamb, goat, rabbit, wild game, chicken, duck, or other birds, liver, kidney, heart, or other organ meats?<br><br>F. Any eggs?<br><br>G. Any fresh or dried fish or shellfish?<br><br>H. Any foods made from beans, peas, lentils, or nuts?<br><br>I. Any cheese, yogurt, milk, or other milk products?<br><br>J. Any foods made with oil, fat, or butter?<br><br>K. Any sugar or honey?<br><br>L. Any other foods, such as condiments, coffee, or tea?<br><br>M. None of the above	A……………………………………|___| <br>B……………………………………|___| <br>C……………………………………|___| <br>D……………………………………|___| <br>E……………………………………|___| <br>F……………………………………|___| <br>G……………………………………|___| <br>H……………………………………|___| <br>I……………………………………|___| <br>J……………………………………|___| <br>K……………………………………|___| <br>L……………………………………|___| <br>M…………………………………|___|	

SECTION 7: Decision-making and women's autonomy

Now, I would like to talk about decision-making in your household.

No.	Question	Response	Code
701.	When you do earn money, who usually decides how the money you personally earn will be used?	1 = Respondent<br>2 = Husband/partner<br>3 = Jointly (with partner or someone else in household)<br>4 = Someone else ____________<br>5 = Respondent never has own earnings	
702.	Would you say that the money you earn is more than, less than, or equal to what your husband/partner earns?	1 = More than him<br>2 = Less than him<br>3 = Equal to him<br>4 = Husband has no earnings<br>5 = Respondent never has own earnings	if Q312=1

		6 = No husband/partner<br>88 = DK/Refused<br>99 = Other ___________________	
703.	Who usually decides how your husband/partner's earnings will be used?	1 = Respondent<br>2 = Husband/partner<br>3 = Jointly (with partner or someone else in household)<br>4 = Someone else _____________<br>5 = No husband/partner	if Q312=1
704.	Who usually makes decisions about major household purchases?	1 = Respondent<br>2 = Husband/partner<br>3 = Jointly (with partner or someone else in household)<br>4 = Someone else _____________	
705.	Who usually makes decisions about health care for yourself?	1 = Respondent<br>2 = Husband/partner<br>3 = Jointly (with partner or someone else in household)<br>4 = Someone else _____________	
706.	Who usually makes decisions about health care for your children?	1 = Respondent<br>2 = Husband/partner<br>3 = Jointly (with partner or someone else in household)<br>4 = Someone else _____________	If Q212 > 0
707.	Who usually makes decisions about visits to your family or relatives?	1 = Respondent<br>2 = Husband/partner<br>3 = Jointly (with partner or someone else in household)<br>4 = Someone else _____________	
	Are you usually allowed to go to the following by yourself, only if accompanied, or not at all?		
708.	The local market to shop	0 = Not at all<br>1 = Only if accompanied<br>2 = By herself	
709.	The local health center	0 = Not at all<br>1 = Only if accompanied<br>2 = By herself	
710.	Visit with friends nearby	0 = Not at all<br>1 = Only if accompanied<br>2 = By herself	
711.	The nearest church/temple/place of worship	0 = Not at all<br>1 = Only if accompanied<br>2 = By herself	
712.	Out of town to visit relatives/friends	0 = Not at all<br>1 = Only if accompanied<br>2 = By herself	

SECTION 8: Group membership & social support

No.	Question	Response	Code

801.	In the past 6 months, which of the following groups existed in your community? In which did you personally participate?		
802.	Agricultural/livestock/fishery group	0 = Group did not exist<br>1 = Group existed but she did not participate<br>2 = Respondent participated in group<br>88 = DK/Refuse	
803.	Business associations/cooperatives	0 = Group did not exist<br>1 = Group existed but she did not participate<br>2 = Respondent participated in group<br>88 = DK/Refuse	
804.	Savings and lending groups	0 = Group did not exist<br>1 = Group existed but she did not participate<br>2 = Respondent participated in group<br>88 = DK/Refuse	
805.	Microcredit (formal or informal)	0 = Group did not exist<br>1 = Group existed but she did not participate<br>2 = Respondent participated in group<br>88 = DK/Refuse	
806.	Women's or mother's group	0 = Group did not exist<br>1 = Group existed but she did not participate<br>2 = Respondent participated in group<br>88 = DK/Refuse	
807.	HIV-specific support groups	0 = Group did not exist<br>1 = Group existed but she did not participate<br>2 = Respondent participated in group<br>88 = DK/Refuse	
808.	Other Health groups	0 = Group did not exist<br>1 = Group existed but she did not participate<br>2 = Respondent participated in group<br>88 = DK/Refuse	
809.	Religious group (any)	0 = Group did not exist<br>1 = Group existed but she did not participate<br>2 = Respondent participated in group<br>88 = DK/Refuse	
810.	Community political group	0 = Group did not exist<br>1 = Group existed but she did not participate<br>2 = Respondent participated in group<br>88 = DK/Refuse	
811.	Are there any other community groups that you have participated in in the last 6 months?	1 = Yes [fill-in]<br>0 = No	
	Now I'd like to discuss how the people you spend time with support you.		
812.	Is there a special person who is around when you are in need?	1 = Strongly disagree<br>2 = Mildly disagree<br>3 = Neutral<br>4 = Mildly agree<br>5 = Strongly agree<br>88 = DK/Refuse	
813.	Is there a special person with whom you can share your joys and sorrows?	1 = Strongly disagree<br>2 = Mildly disagree<br>3 = Neutral<br>4 = Mildly agree<br>5 = Strongly agree	

		88 = DK/Refuse	
814.	Does your family really try to help you?	1 = Strongly disagree<br>2 = Mildly disagree<br>3 = Neutral<br>4 = Mildly agree<br>5 = Strongly agree<br>88 = DK/Refuse	
815.	Do you get the emotional help and support you need from your family?	1 = Strongly disagree<br>2 = Mildly disagree<br>3 = Neutral<br>4 = Mildly agree<br>5 = Strongly agree<br>88 = DK/Refuse	
816.	Do you have a special person who is a real source of comfort to you?	1 = Strongly disagree<br>2 = Mildly disagree<br>3 = Neutral<br>4 = Mildly agree<br>5 = Strongly agree<br>88 = DK/Refuse	
817.	Do your friends really try to help you?	1 = Strongly disagree<br>2 = Mildly disagree<br>3 = Neutral<br>4 = Mildly agree<br>5 = Strongly agree<br>88 = DK/Refuse	
818.	Can you count on your friends when things go wrong?	1 = Strongly disagree<br>2 = Mildly disagree<br>3 = Neutral<br>4 = Mildly agree<br>5 = Strongly agree<br>88 = DK/Refuse	
819.	Can you talk about your problems with your family?	1 = Strongly disagree<br>2 = Mildly disagree<br>3 = Neutral<br>4 = Mildly agree<br>5 = Strongly agree<br>88 = DK/Refuse	
820.	Do you have friends with whom you can share your joys and sorrows?	1 = Strongly disagree<br>2 = Mildly disagree<br>3 = Neutral<br>4 = Mildly agree<br>5 = Strongly agree<br>88 = DK/Refuse	
821.	Is there a special person in your life who cares about your feelings?	1 = Strongly disagree<br>2 = Mildly disagree<br>3 = Neutral<br>4 = Mildly agree<br>5 = Strongly agree<br>88 = DK/Refuse	
822.	Is your family willing to help you make decisions?	1 = Strongly disagree<br>2 = Mildly disagree<br>3 = Neutral	

		4 = Mildly agree<br>5 = Strongly agree<br>88 = DK/Refuse	
823.	Can you talk about your problems with your friends?	1 = Strongly disagree<br>2 = Mildly disagree<br>3 = Neutral<br>4 = Mildly agree<br>5 = Strongly agree<br>88 = DK/Refuse	

SECTION 9: Exit Interview (Endline Only)

Instructions: Present the woman with her records and discuss. Use this time to clarify balances, confirm outliers, examine discrepancies as noted in the woman's diary file. After all are complete, ask the following:

901. How would you describe your experience of keeping this diary over the past 7 months?

902. Has your thinking about money changed at all from keeping the diary? If so, how?

903. Have your spending habits changed at all from keeping the diary? If so, how?

904. As we design a new program to help HIV-positive pregnant women and new mothers manage their finances to improve retention in PMTCT, what do you think would be most helpful to you? *Probe:* Specific ideas for savings and loans programs

905. Do you have any final thoughts to share about how money comes and goes in your life that you think would be helpful for us to know about?
