# Supplementary material for: Using financial diaries to understand the economic lives of HIV-positive pregnant women and new mothers in PMTCT in Zomba, Malawi
Source: PLoS One. 2021 Jul 30;16(7):e0252083. doi: 10.1371/journal.pone.0252083 (PMC8323884; doi:10.1371/journal.pone.0252083)
Supplement: S2 File — (PDF) [file pone.0252083.s002.pdf]

FHI 360

Mafunso antchito zokhudza mabukhu a zandalama
Version 2.0; May 29, 2018

**Mutu:**	*Mabuku a zandalama pofuna kumvetsa nkhani za kusunga ndi kulondoloza ndalama za azimayi oyembekezera komanso amene ali ndi ana akhanda mu boma la zomba mu Malawi*
**Wothandiza :**	*FHI 360 and USAID*
**Keyala :**	*FHI 360, 359 Blackwell St, Suite 200, Durham, NC 27701 USA*

Zofunika kutsata wochita kafukufuku:

No.	Question	Response	Code
Int	Interviewer ID	[fill-in]	
Today	Date of interview	*[calendar automated to mark system date, but can be modified by data collector]*	
Site	Site/Location	0 = Likangala Health Centre (Rural)<br>1 = Pirimiti Community Hospital (Peri-urban)<br>2 = Matawale Health Centre (Urban)	
PID1	Participant ID	[fill-in]<br><br>*Note to programmer:*<br>*1001-1200 only valid if site=0*<br>*2001-2200 only valid if site=1*<br>*3001-3200 only valid if site=2*	

<u>GAWO 1: Zofunika woyankha</u>

Ndikufuna kuti ndiyambe ndi kupeza mbiri yanu

No.	Funso	Yankho	Nambala
101.	Muli ndi zaka zingati?	[fill-in, integer only]	constraint 18-49
102.	Moyo wanu wa banja ndi uti?	1 = Wokwatiwa<br>2 = Wapa mitala<br>3 = Wosakwatiwa<br>4 = Wosudzulidwa<br>5 = Wopatukana<br>6 = Namfedwa<br>7 = Bwenzi<br>99 = Zina__________________	
103.	Kodi munaphunzirako sukulu?	0 = Ayi<br>1 = Inde	
104.	Sukulu yanu mudalekezera kalasi liti?	1 = Pulayimale<br>2 = Fomu 1 ndi Fomu 2<br>3 = Fomu 3 ndi Fomu 4	if Q104=1

		4 = Sukulu ya za malonda<br>5 = Yunivesite<br>99 = Zina__________________	
105.	Kodi mumatha kuwerenga?<br>*(Wofunsa mafunso awonetse mayiyu khadi la mawu oti aweerenge)*<br><br>*Ndimagula chimanga kwa ogulitsa mumsika*	1 = Sangawerenge zonse chiganizo chonse<br>2 = Angathe kuwerenga mbali ina ya chiganizo<br>3 = Angathe kuwerenga chiganizo chonse<br>4 = Ali ndi khungu/ sawonetsetsa	
106.	Kodi chipembedzo chanu ndi chiti?	1 = Chikhirisitu<br>2 = Chisilamu<br>3 = Chipembedzo cha makolo<br>4 = Alibe chipembedzo<br>99 = Zina _______________	
107.	Kodi mtundu wanu ndi chiyani?	1 = Chewa<br>2 = Lomwe<br>3 = Yao<br>4 = Ngoni<br>5 = Tumbuka<br>6 = Sena<br>7 = Tonga<br>8 = Nyanja<br>99 = Zina _______________	

GAWO 2: Kakhalidwe ka pabanja

Tsopano tikamba za pabanja panu

No.	Funso	Yankho	Nambala
201	Ndikufuna ndilembere kuchuluka kwa anthu amene amakhala pa banja panu. Banja ndi gulu la anthu amene amakhala pamodzi, kupeza ndalama ndi kudya chakudya pamodzi tsiku lililonse.<br><br>Pokhala ndi maganizo amenewo, kodi banja lanu lili ndi anthu angati?	A. Asanu ndi awiri kapena kupitilirako<br>B. Asanu ndi m'modzi<br>C. Asanu<br>D. Anayi<br>E. M'modzi, awiri, atatu	
201a	Mutu wa banja ndi wa mwamuna kapena wa mkazi?	0 = Mwamuna<br>1 = Mkazi	

201.	Kodi (wachikulire) mayi kapena mkazi amatha kulemba ndi kuwerenga chingerezi kapena Chichewa? Ngakhale makhomo ambiri atha kukhala ndi Mzimayi mmodzi mutu wa banja, ena ali ndi oposera mmodzi kukhala mutu wa banja. Ngati zili choncho tikufunsa, **mzimayi wamkulu amene ali mutu wa banja. Wamkulu amene ali mutu wa banja atha kusakhala amene ali wamkulu kwambiri pakhomopo (Chisanzo, agogo atha kukhala pakhomopo koma sali mutu wa banja).**	A. Ayi<br>B. Eya Chichewa chokha<br>C. Eya, chingerezi (mosalabadira Chichewa)<br>D. Palibe mayi / mkazi wachikulire pa banjali	
202.	Kodi pansi pa nyumba yogona kwenikweni panapangidwa ndi zipangizo ziti?	A. Yozira ndi mtsiro kapena mchenga<br>B. Yasimenti, matabwa, matayilosi kapena zina	
203.	Kodi khoma la kunja kwa nyumba yogona linapangidwa ndi zipangizo zotani?	A. Yomata kapena yamaudzu<br>B. Yazidina<br>C. Yamdindo, njerwa zootcha, simenti, malata kapena zina	
204.	Kodi denga la nyumba yaikulu linapangidwa ndi zipangizo ziti?	A. Udzu, pepala la pulasitiki. Kapena zina<br>B. Malata, matayilosi owumba kapena simenti	
205.	Kodi muli ndi chimbudzi cha mtundu wanji pa khomo panu?	A. Palibe, chokumba koma chosafolera chogwiritsidwa ndi mabanja ena, zina.<br>B. Chokumba koma chosafolera chapabanja lathu lokha.<br>C. Chokumba komaso chofolera chogwiritsidwa ntchito ndi mabanja ena<br>D. Chokumba chili chofolera chogwiritsidwa ntchito ndi banja lathu lokha, chokumba komanso chasimenti kapena chamadzi	

206.	Kodi banjali limagwiritsa ntchito moto wamtundu wanji?	A. Nkhuni zokatola, nkhumi zogula, udzu kapena gasi<br>B. Parafini kapena zina<br>C. Batire, tochi, makandulo kapena magetsi	
207.	Kodi anthu apa banjali amagona mu neti kuti atetezeke ku udzudzu chaka chonse?	A. Ayi<br>B. Inde	
208.	Kodi pabanja panu mumagwiritsa ntchito matebulo?	A. Ayi<br>B. Inde	
209.	Kodi banjali lili ndi ma bedi?	A. Ayi<br>B. Inde	
210.	Kodi m'banjali ana osakwana zaka 18 alipo angati?	Ana___ ___ osafika zaka 18	constraint: 0-20
211.	Pa ana amenewo, ndi angati amene amadalira inu pachilichonse?<br>Apa tikutanthauza ana amene amadalira in upa chisamaliro chawo cha tsiku ndi tsiku.	Ana___ ___	constraint: 212=<211

Gawo 3: Kapezedwe ka chuma ndi kasungidwe ka chuma

Pa mafunso otsatirawa, ndikufuna ndiyankhule nanu za momwe mumapezera ndalama pa banja panu, makamaka pandalama ndi katundu amene inu mumalamulira.

No.	Funso	Yanko	Namabal a
301.	Kodi muli ndi malo anuanu?	0 = Ayi<br>1 = Inde, koma a anthu angapo<br>2 = Inde a ine ndekha	
302.	Kodi muli ndi ziweto zanuzanu?	0 = Ayi<br>1 = Inde koma za anthu angapo<br>2 = Inde za ine ndekha	
303.	Kodi muli ndi foni ya m'manja yanuyanu?	0 = Ayi<br>1 = Inde koma ya anthu angapo<br>2 = Inde yangayanga	
304.	Kodi muli ndi nyumba yanuyanu?	0 = Ayi<br>1 = Inde ya anthu angapo<br>2 = Inde yangayanga	
	304 a. Kodi muli ndi nyumba kapena malo amene mumapangapo lendi mwa inu nokha?	0 = Ayi<br>1 = Inde koma ya anthu angapo<br>88 = Inde yangayanga	
	304b. Kodi muli ndi akaounti yanuyanu ku ma bank akuluakulu	0 = Ayi<br>1 = Inde<br>88 = DK/Refuse	

305.	Kodi mwagwirako ntchito pofuna kupeza ndalama masiku 30 apitawa ?	0 = Ayi → **pitani pa 310**<br>1 = Inde	
306.	Kodi munkagwira ntchito yanji kuti mupeze ndalama masiku 30 apitawa?<br><br>**Sankhani chimodzi choyenera pa gulu la ntchito zatchulidwazi**	1 = Yaulimi<br>2 = Yausodzi<br>3 = Ogwira ntchito za umoyo<br>4 = Ya zamanja zophunzira kapena luso lobadwa nalo<br>5 = Yazapakhomo<br>6 = Yauphunzitsi<br>7 = Yazamalonda<br>8 = Ntchito za mu ofesi<br>99 =Zina________________	
307.	Kodi ntchito yanuyo pa masiku 30 mungaifotokoze bwanji?	1 = Yokhazikika<br>2 = Ganyu<br>3 = Yapakanthawi<br>4 = Ganyu wolandira pa tsiku<br>5 = Yodzilemba<br>99 = zina________________	
308.	Kodi mumapeza pafupifupi ndalama zingati pa masiku 30, mu ma kwacha?<br><br>Ngati wotenga nawo mbali sakudziwa ikani 88	__________________ kwacha<br>88 = DK/Refused	
309.	Kodi mumalandila malipiro anu motani?	1 = Tsiku lililonse<br>2 =Pasabata<br>3 =Pamwezi<br>4 = Pakanthawi<br>5 = Sizidziwika kwenikweni<br>99 =Zina ________________	
310.	Ngati simunagwireko ntchito masiku 30 apitawa, kodi mudagwirako ntchito m'chaka chathachi?	0 = Ayi → **Pitani pa 312**<br>1 = Inde	relevance : Q305 is NOT 1
311.	Kodi ntchito imene munkagwira chaka chatha inali yotani?<br><br>**Sankhani chimodzi choyenera pa ntchito zatchulidwa**	1 = Yaulimi<br>2 = Yausodzi<br>3 = Ya zaumoyo<br>4 = Ntchito za manja za luso ndi zo phunzira<br>5 = Ya pakhomo<br>6 = Yauphunzitsi<br>7 = Yazamalonda<br>8 = Ntchito zamuofesi<br>99 = Zina ________________	relevance : Q305 is NOT 1
	311ª Kuphatikizapo ndi inu, ndi anthu angati amene amapeza ndalama zomwe zimathandizira pakhomo pano	Integer fill-in	
312.	Kodi muli ndi mwamuna kapena bwenzi?	0 = Ayi → **Pitani pa 316**<br>1 = Inde	

	312ª Kodi mwamuna kapena bwenzi lanulo likukhala limodzi ndi inu?	0 = Ayi<br>1 = Inde	relevance : if 312=1
313.	Kodi mwamuna/bwenzi lanu lagwirako ntchito masiku 30 apitawa	0 = Ayi → **Pitani pa 316**<br>1 = Inde<br>88 = DK/Refused	
314.	Kodi ankagwira ntchito yanji kuti apeze ndalama pa masiku 30 apitawa?<br><br>**Sankhani chimodzi choyenera pa ntchito zatchulidwa**	1 = Yaulimi<br>2 = Yausodzi<br>3 = Ya zaumoyo<br>4 = Ntchito za manja za luso ndi zo phunzira<br>5 = Ya pakhomo<br>6 = Yauphunzitsi<br>7 = Yazamalonda<br>8 = Ntchito zamuofesi<br>99 = Zina	
315.	Kodi ntchito yawo yeniyeni mungaifotokoze motani?	1 = Yokhazikika?<br>2 = Ganyu<br>3 = Yapakanthawi<br>4 = Ganyu wolandira patsiku<br>5 = Yodzilemba<br>88 = DK/Refused<br>99 = Zina ________________	
316.	Kodi aliponso wina amene amapeza ndalama zothandizira pa banja lanu?<br>Ngati pali anthu angapo amene amapeza ndalama kuthandizila pakhomopo, sankhani amene akuthandizila kwambiri	0 = Ayi → **Pitani pa 320**<br>1 = Inde	
317.	Kodi ameneyo ndi ndani wanu?	1 = Kholo<br>2 = Mwana<br>3 = Wachibale<br>4 = Amalume kapena azakhali<br>5 = Mphwanga kapena mdzukulu<br>99= zina ________________	
318.	Kodi munthuyo amagwira ntchito yanji kuti apeze ndalama pa masiku 30 apitawo?<br><br>**Sankhani chimodzi choyenera pa ntchito zatchulidwa**	1 = Yazaulimi<br>2 = Yausodzi<br>3 = Yokonza ndi kugulitsa<br>4 = Yazaluso ndi za malonda<br>5 = Yazomangamanga<br>6 = Yazumoyo<br>7 = Yauphunzitsi<br>8 = Ntchito zina<br>88 = DK/Refused<br>99 = Zina ________________	

319.	Kodi ntchito ya munthuyo mungaifotokoze bwanji?	1 = Yokhazikika<br>2 = Ganyu<br>3 = yapakanthawi<br>4 = Yolandira pa tsiku<br>5 = Yodzilemba<br>88 = DK/Refused<br>99 = zina________________	
320.	Ndi ndani amayang'anira chuma chapakhomo panu?	1 = Woyankha mafunsoyu<br>2 = Mamuna/bwenzi<br>3 = Onse mothandizana<br>99 = zina__________________	
321.	Kodi banja lanu mumatsatira ndondomeko ya ndalama (budget} pa masiku 30 apitawa?	0 = Ayi → **pitani pa 324**<br>1 = Inde<br>88 = DK/Refused	
322.	Kodi zinthu zogulidwa zinali zoposera ndondomeko ya ndalama zanu pa masiku 30 apitawa?	0 = Ayi → **Pitani pa 324**<br>1 = Inde<br>88 = DK/Refused → **Pitani pa 324**	
323.	Ndi zinthu ziti zomwe zidagulidwa, zomwe sizidali nawo pa bajeti kapena kut pa ndondomeko ya ndalama zanu?<br><br>Lembani zinthu kapena thandizo , osati ndalama, zimene zinali kunja kwa ndondomeko ya za chuma (Budget) mchingerezi	(Text fill in)	
324.	Kodi mudali ndi cholinga chosunga ndalama pa masiku 30 apitawo	0 = Ayi → **Pitani pa 326**<br>1 = Inde	
325.	Kodi mudakwaniritsa cholinga chanu kumasunga ndalama pa masiku 30 apitawa?	0 = Ayi<br>1 = Inde → **Pitani pa 327**	
326.	Kodi mudasungako ndalama zina zilizonse pa masiku 30 apitawa?	0 = Ayi<br>1 = Inde	
327.	Kodi pano mwatsala ndi ndalama zingati pa zomwe mudasunga, mumakwacha, kuphatikiza ndalama zimene munasunga pa masiku opitilira 30 m'mbuyomu?<br>Ngati wotenga mbali sakudziwa lembani 88	_________________kwacha<br>88 = DK/Refused	
328.	Kodi muli ndi ngongole zimene simunabweze?	0 = Ayi → **Pitani pa gawo 4**<br>1 = Inde<br>88 = DK/Refused	
329.	Kodi mudatengako ngongole pa masiku 30 kapena kupitilirapo?	0 = Ayi → **Pitani pa 332**<br>1 = Inde	

330.	Kodi ngongole zimene mudatenga masiku 30 apitawa ndi zingati zonse pamodzi muma kwacha?<br>Ngati wotenga mbali sakudziwa lembani 88	_________________ kwacha<br>88 = DK/Refused	
331.	Kodi ngongolezo ndi mbali imodzi ya ndondomeko yanu ya ndalama (budget)?	0 = Ayi<br>1 = Inde	relevance : skip if Q321=0 or 88
332.	Kodi muli ndi ngongole zingati mumakwacha, kuphatikiza zimene munkatenga masiku opitilira 30 m'mbuyomu?<br>Ngati wotenga mbali sakudziwa lembani 88	_________________ kwacha<br>88 = DK/Refused	

GAWO 4: Za Chipukuta misonzi pa chuma ndi phindu

Pa mafunso otsatirawa, ndikufuna ndikambe nanu za zimene mumapindula kapena mumataya

No.	Funso	Yankho	Nambala
401.	Poyamba tiyeni tikambirane za zimene munatayako pa banja lanu pamiyezi isanu ndi umodzi yapitayi. Ndiwerenga mndandanda wa mavuto ndipo pamene ndikuwerenga inu muzindidziwitsa ngati vuto limenelo munakumanako nalo .<br><br>*Sankhani zonse zimene munakumanako nazo*		
	A. Kuononga ndalama zochuluka pakupeza chithandizo cha mankhwala kapena maliro kamba ka mmodzi amene amakhala pakhomopo<br>B. Kuonongeka kwa ndalama za pa khomo kamba koti mmodzi mwa anthu apa khomo anadwalika kwambiri or anamwalira<br>C. Kumwalira kwa munthu amene amathandiza kupeza chuma pa banjali<br>D. Kuchotsedwa ntchito kwa munthu amene amathandiza kupeza chuma pa banjali<br>E. Kulephera kwa bizinezi kwa munthu wina aliyense wa pa banja lanu<br>F. Kuchoka pakhomo kwa mmodzi wa banjali chifukwa cha banja, kulekakana. Kapena kungosiyidwa.<br>G. Panali chiononongeko cha katundu chifukwa cha akuba, umbanda, kapena ngozi ya moto<br>H. Mbeu za pa banjali zinakanika<br>I. Panali kuonongeka kwa zinthu chifukwa cha ngozi zadzidzidzi (kukanika kwa mbeu, kufa kwa ziweto ndi zina)<br>J. Banjali linakumana ndivuto chifukwa cha kutsika mtengo kwa mbeu<br>K. Kukonzetsa nyumba kapena katundu wa munyumba<br>L. Ziopsezo zina<br>M. Palibe		
402.	Tsopano tikambirane za momwe banjali lapezera thandizo pa miyezi isanu ndi umodzi yapitayi, kodi alipo wina m'banjali amene:<br><br>*Sankhani zonse zimene zili zoona*		
	A. Anapeza ntchito yatsopano<br>B. Anayamba kulandira chithandizo cha ndalama kuchokera ku boma kapena ku mabungwe amane sali a boma?<br>C. Analandira chuma chochuluka, ngati mphatso, anapambana mpikisano wina wake, kapena malowolo ndi zina?		

	D. Anapeza thandizo la maphunziro<br>E. Munalandira thandizo lochuluka<br>F. Anapeza phindu lalikulu pa banjali kapena pa zaulimi chifukwa cha kukwera mitengo kwa mbeu<br>G. Zina (Lembani)<br>H. Palibe	

GAWO 5: Zakupewa kufalikira kwa kachirombo kuchoka kwa mayi kupita kwa mwana (PMTCT Experience)

Tsopano ndikufuna kuti tilankhule zokhudza kupeza thandizo pofuna kupewa kupatsira kachirombo ka HIV kuchokera kwa mayi kupita kwa mwana.

No.	Funso	Yankho	Nambala
	**MFUNDO ZAZIKULU**		
501.	Kodi mudaulula kwa amuna anu kuti muli ndi kachirombo ka HIV?	0 = Ayi<br>1 = Inde<br>2 = Ndilibe mamuna<br>88 = DK/Refused	
502.	Kodi mudaulula kwa anzanu kapena achibale kuti muli ndi kachilombo ka HIV?	0 = Ayi<br>1 = Inde<br>88 = DK/Refused	
503.	Kodi panopa ndinu woyembekezera?	0 = Ayi → **Pitani pa 505**<br>1 = Inde	
504.	Panopa pakati panu ndi papakulu bwanji?<br><br>Ngati wotenga nawo mbali sakudziwa, lembani 88	Masabata ___ ___ Pitani pa → **506**<br>88 = DK/Refused → **Pitani pa 506**	constraint: 1-45
505.	Kodi mwana wanu omalizira anabadwa liti?	1 = Masiku apitawo<br>7 = Masabata apitawo<br>30 = Miyezi yapitayo	if Q503=0
	505ª Ndi masiku/masabata/miyezi ingati yapitayo pamene mwana wanu anabadwa?	(fill iin integer)	if Q503=0
	**Zakuyezetsa**		
506.	Kodi munkadziwa kuti muli ndi kachirombo musanatenge kapena mutatenga pakati muli napopa?	1 = Ndisanatenge pakati<br>2 = Nditatenga kale pakati	
507.	Kodi ndi chaka chani pamene adakupezani ndi kachirombo ka HIV? *Yesetsani kuthandizila otenga nawo mbali akumbukire mwezi kapena chaka chimene anapezeka ndi kachilombo. Ngati sakuthata kukumbukira, afunsensi aganizire. Ngati sakudziwa, lembani 8888*	(Fill in integer)	constraint: 1985-2019 OR 8888
	507a Kodi ndi chaka chani pamene adakupezani ndi kachirombo ka HIV	1 = January<br>2 = February	constraint: 1-12 or 88

	Yesetsani kuthandizila otenga nawo mbali akumbukire mwezi kapena chaka chimene anapezeka ndi kachilombo. Ngati sakuthata kukumbukira, afunsensi aganizire.<br>*Ngati sakudziwa, lembani 8888*	3 = March<br>4 = April<br>5 = May<br>6 = June<br>7 = July<br>8 = August<br>9 = September<br>10 = October<br>11 = November<br>12 = December<br>88 = Don't know	
508.	Kodi munayesedwa za HIV ngati mbali imodzi yachisamaliro cha pakati muli napopa?	0 = Ayi<br>1 = Inde<br>88 = DK/Refused	relevance: ask if Q506=2
	**Musanayambe ndondomeko yopewera kufalikira kwa kachirombo kuchokera kwa mayi kupita kwa mwana**		
509.	Kodi apa ndi pakati/ mwana woyamba?	0 = Ayi<br>1 = Inde → **Pitani pa 511**	
510.	*Ngati ayi pa funso 509 ndipo munayezetsa ndi kupezeka ndi kachirombo ka HIV musanakahale ndi pakati pamenepa (funso 506):*<br><br>Kodi mudagwiritsako ntchito ndondomeko yopewera kufalikira kwa kachirombo kuchoka kwa mayi kupita kwa mwana pakuyembekezera kulikonse kapena kumeneku?	0 = Ayi<br>1 = Inde<br>88 = DK/Refused	relevance: ask if Q509=0 AND Q506=1
	**Za mankhwala otalikitsa moyo (Martenal ART)**		
511.	Kodi nthawi ina yake mudamwako mankhwala otalikitsa moyo pofuna kupewa kupatsira mwana kachirombo pa kuyembekezeraku?	0 = Ayi → **Pitani pa 514**<br>1 = Inde	
512.	Kodi mudayamba chaka chiti kumwa mankhwalawa?<br>*Yesetsani kuthandizila otenga nawo mbali akumbukire mwezi kapena chaka chimene anayamba kumwa mankhwala otalikitsa moyo. Ngati sakuthata kukumbukira, afunseni aganizire.*<br>*Ngati sakudziwa, lembani 8888*	[fill in integer)	relevance: if Q511=1<br><br>Constraint:<br>>=Q507<br>=<2019<br>OR 8888
5<br>5	512a Ndi mwezi wanji umene mudayamba kumwa mankhwala otalikitsa moyo?	(fill in integer)	relevance:<br>if Q511=1<br><br>constraint<br>1-12 OR 88

	Yesetsani kuthandizila otenga nawo mbali akumbukire mwezi kapena chaka chimene anayamba kumwa mankhwala otalikitsa moyo. Ngati sakuthata kukumbukira, afunseni aganizire.<br>*Ngati sakudziwa, lembani 88*		
513.	Kodi munayamba kumwa makhwalawa nthawi yomweyo mutangopezeka ndi kachirombo ka HIV?	0 = Ayi<br>1 = Inde	if Q511=1
514.	Kodi munayamba mwasiyako kumwa mankhwalawa muli ndi mimba chomwechi?	0 = Ayi → **Pitani516**<br>1 = Inde	Q503=1
5514 a	Kodi munayamba mwasiyako kumwa mankhwalawa muli ndi mimba kapena mukuyamwitsa mwana wanu?	0 = Ayi → **Pitani 516**<br>1 = Inde	
515.	Mudasiyiranji?<br><br>*Chidziwitso kwa ofunsa mafunso: Mumulole woyankhayu kuyankha momasuka, kenako musankhe yankho loyenera*	1 = Kusasangalatsidwa<br>2 = Kutangwanika, kupanga kaye zinthu zina zaphindu<br>3 = Mankhwalawa amandidwalitsa<br>4 = Ndikudzimva kuti ndili wa thanzi, mankhwalawa safunikanso<br>5 =Ndilibe chakudya chokwanira chakudya ndikamwa mankhwala<br>6 =Ndalama yoyendera idakwera<br>7 = Kuchipatala ndi kutali<br>8 = Mwamuna wanga sandilimbikitsa<br>9 = Zovuta zimene zilipo kuchipatala ndi anthu ogwira ntchito kumeneko<br>10 = Kukana kusalidwa<br>88 = DK/Refused<br>99 = Zina________________	
516.	Kodi panopa mukumamwa mankhwala otalikitsa moyo	0 = Ayi<br>1 = Inde → **Pitani pa 518**	
517.	Chifukwa chiyani?<br><br>*Chidziwitso kwa ofunsa: Mulole woyankha ayankhe mwaufulu ndi musankhe yankho loyenera*	1= Kusasangalatsidwa<br>2 = Kutangwanika, kupanga kaye zinthu zina zaphindu<br>3 = Mankhwalawa amandidwalitsa<br>4 = Ndikudzimva kuti ndili wa thanzi, mankhwalawa safunikanso<br>5 =Ndilibe chakudya chokwanira chakudya ndikamwa mankhwala<br>6 =Ndalama yoyendera idakwera<br>7 = Kuchipatala ndi kutali<br>8 = Mwamuna wanga sandilimbikitsa<br>9 = Zovuta zimene zilipo kuchipatala ndi anthu ogwira ntchito kumeneko<br>10 = Kukana kusalidwa	

		88 = DK/Refused<br>99 = Zina ________________	
518.	Kodi munasemphanako ndi thandizo kapena munalepherako kupita kukalandira mankhwala okhudza ndondomeko zopewera kufalikira kwa kachirombo kuchokera kwa mayi kupita kwa mwana (PMTCT) pamene muli/munali oyembekezera chomwechi?	0 = Ayi **pitani pa 520 kapena 521 (onani funso 503)**<br>1 = Inde, ndinalepherako kupita<br>2 = Inde , ndinasemphanako ndi thandizo<br>3 = Inde, zonse	
519.	Kodi ndi zifukwa ziti/chifukwa chiti chinapangitsa kusemphana ndi thandizo kapena kupita kokalandira mankhwala? (sankhani zonse zoyenera)<br><br>*Chidziwitso kwa ofunsa: Mulole woyankha ayankhe mwaufulu ndi musankhe yankho loyenera*	1= Kusasangalatsidwa<br>2 = Kutangwanika, kupanga kaye zinthu zina zaphindu<br>3 = Mankhwalawa amandidwalitsa<br>4 = Ndikudzimva kuti ndili wa thanzi, mankhwalawa saufunikanso<br>5 =Ndilibe chakudya chokwanira chokudya ndikamwa mankhwala<br>6 =Ndalama yoyendera idakwera<br>7 = Kuchipatala ndi kutali<br>8 = Mwamuna wanga sandilimbikitsa<br>9 = Zovuta zimene zilipo kuchipatala ndi anthu ogwira ntchito kumeneko<br>10 = Kukana kusalidwa<br>88 = DK/Refused<br>99 = Zina ________________	if Q518=1,2 OR 3
	**Za amayi oyembekezera okha (Onani funso 503)**		
520.	Mukukonzekera kumadzayamwitsa mwana wanu akadzabadwa?	0 = Ayi<br>1 = Inde<br>88 = DK/Refused<br>**Pa mayankho onse, Pitani Gawo 6**	relevance: Q503=1
	**ZA AMAYI AMENE AYAMBA KUBEREKA KUMENE (Onani funso Za 503)**		
521.	Kodi mwana wanu ndi wamsinkhu wanji?	Masiku___ ___ → **pitani pa 523**<br>Masabata___ ___ → **pitani pa 523**<br>Miyezi___ ____ → **Skip to 523**<br><br>999 =Mwana anamwalira	relevance Q503=0
521 a	Mwana wanu ali ndi masiku/masabata/miyezi ingati?	(fill in integer)	if Q521=1, 7 or 30
522.	Kodi mwanu anamwalira ali pa msinkhu wanji?	1 = Masiku___ ___<br>7 = Masabata___ ___<br>30 = Miyezi___ ___	relevance Q521=999

		999 = Mwana anamwalira nthawi yobadwa → **Pitani pa Gawo 6 (endline ONLY)**<br><br>**Mafunso oyambirira athera pamenepa,**	
522a	Anali ndi masiku/miyezi/zaka zingati pamene mwana wanu anamwalira?	(fill in integer)	
523.	Kodi mwana wanu anayamba kumwa mankhwala otalikitsa moyo atangobadwa?	0 = Ayi → **Pitani pa 526**<br>1 = Inde	relevance Q503=0<br><br>Q522!=999
524.	***Kutengera yankho la pa 521.***<br>***Ngati mwana ali wochepera masabata 6, pitani pa funso 529***<br>***Ngati mwana anamwalira asanafike masabata 6 (onani funso 522), Pitani pa gawo 6***<br><br>(Ngati mwana ndi wopitirira masabata 6)<br>Kodi mwana wanu anapitiliza kumwa mankhwalawa masabata onse 6 ?	0 = Ayi<br>1 = Inde → **Pitani pa 526**	relevance Q503=0<br><br>Q523=1<br><br>babyage >=42<br>OR<br>deathage >=42
525.	Kodi chifukwa cheni cheni chimene munasiyira ndi chiyani?<br><br>*Chidziwitso kwa ofunsa: Mulole woyankha ayankhe mwaufulu ndi musankhe yankho loyenera*	1= Kusasangalatsidwa<br>2 = Kutangwanika, kupanga kaye zinthu zina zaphindu<br>3 = Mankhwalawa ali ndi zotsatira zosakhara bwino<br>4 = Ndikudzimva kuti ndili wa thanzi, mankhwalawa saufunikanso<br>5 =Ndilibe chakudya chokwanira chakudya ndikamwa mankhwala<br>6 =Ndalama yoyendera idakwera<br>7 = Kuchipatala ndi kutali<br>8 = Mwamuna wanga sandilimbikitsa<br>9 = Zovuta zimene zilipo kuchipatala ndi anthu ogwira ntchito kumeneko<br>10 = Kukana kusalidwa<br>88 = DK/Refused<br>99 = Zina _________________	relevance Q503=0 AND Q524=0<br><br>Q522!=999
526.	(Ngati mwana ali optirira masabata 6)<br>Kodi mwana wanu anayesedwa atafika masabata 6?	0 = Ayi → **Pitani pa 528**<br>1 = Inde<br>88 = DK/Refused → **Pitani pa 528**	relevance Q503=0<br><br>babyage >=42

			Q522!=999
527.	Kodi mungakonde kugawana nafe zotsatira za mwana wanu momwe adayesedwa HIV?	0 = Alibe kachirombo<br>1 = Ali ndi kachirombo<br>2 = Sindikufuna kuulula<br>3 = Sindikudziwa<br>**Pitani pa 529 za mayankho onsewa**	relevance Q503=0<br><br>Q526=1<br><br>Q522!=999
528.	Kodi mwana wanu sanayesedwe pa masabata 6?<br><br>*Chidziwitso kwa ofunsa: Mulole woyankha ayankhe mwaufulu ndi musankhe yankho loyenera*	1= Kusasangalatsidwa<br>2 = Kutangwanika, kupanga kaye zinthu zina zaphindu<br>3 = Mankhwalawa amamudwalitsa mwana<br>4 = Ndikudzimva kuti mwanayu ali ndi thanzi, mankhwalawa saufunikanso<br>5 =Ndilibe chakudya chokwanira chakudya ndikamwa mankhwala<br>6 =Ndalama yoyendera idakwera<br>7 = Kuchipatala ndi kutali<br>8 = Mwamuna wanga sandilimbikitsa<br>9 = Zovuta zimene zilipo kuchipatala ndi anthu ogwira ntchito kumeneko<br>10 = Kukana kusalidwa<br>11= Mwana adamwalira<br>88 = DK/Refused<br>99= Zina _______________	relevance Q503=0<br><br>Q526!=1<br><br>Q522!=999
529.	Kodi mwanayu amayamwa?	0 = Inde<br>1 = Ayi	relevance Q503=0<br><br>Q521!=999
530.	Kodi mwanayu mumamudyedsa zakudya zina kapena zamadzimadzi osati mkaka wam'mawere?	0 = Ayi → **Pitani pa 533**<br>1 = Inde	relevance Q503=0<br><br>Q521!=999
531.	Kodi ndi zakudya zina ziti zimene mumadyetsa mwana wanu?<br><br>Sankhani zones zimene zatchulidwa	1 = Madzi akumwa okha<br>2 = Juwisi<br>3 = Msuzi wa ndiwo<br>4 = Mkaka wa ufa, wauwisi kapena ochokera ku ziweto<br>5 = Zakudya za wana<br>6 = Yogati<br>7 = Buledi, mpunga, nudozi, phala ndi zakudya zina zochokera ku ufa<br>8 =Zipatso<br>9 = Ndiwo za masamba<br>10 = Nyama<br>99 = Zina__________________	relevance Q530=1

532.	Nthawi imene munamuyambitsa zakudya zimenezi mwana wanu anali ndi zaka zingati?	Masiku____<br>Masabata____<br>Miyezi____<br>88 = DK/Refused	relevance<br>Q503=0<br><br>Q530=1<br><br>Q521!=999
5532 a	Kodi mwana wanu anali ndi masiku/masaba/miyezi ingati pamene mudamuyambisa zakudyazi?	(fill in integer)	Q532=1, 7 or 30
533.	Kodi mudalandira uphungu wa dokotala kapena anamwino wa momwe mungayamwitsire mwana wanu popewa kumupatsira kachirombo?	0 = Ayi<br>1 = Inde	relevance<br>Q503=0<br>AND<br>Q521!=999
534.	Kodi muli ndi mavuto kuyamwitsa mwana wanu?	0 = Ayi → **Pitani ku gawo 6**<br>1 = Inde	relevance<br>Q503=0<br>AND<br>Q521!=999
535.	Mavutowo ndi ati?<br><br>*Sankhani zones zimene zatchulidwa*<br><br>*Chidziwitso kwa ofunsa mafunso: Mulole woyankha kuti ayankhe mwaufulu kenakamusankhe yankho loyenera*	1 = Vuto la mawere (Mawere otupa kwambiri, nkhumbu zazilonda ndi zina)<br>2 = Mawere satulutsa mkaka<br>3 = Ndimaopa kupatsira mwana kachirombo ka HIV<br>4 = Kusafuna kusalidwa<br>5 = Mkamwa mwa mwana muli zironda<br>6 = Kusowa chithandizo kuchokera kwa anthu a pabanja langa<br>7 = Ndimapita kuntchito<br>88 = DK/Refused<br>99 = Zina ___________________	relevance<br><br>Q534=1

GAWO 6 : Chakudya chokwanira ndi kasinthasintha wa zakudya pa banja

Tsopano ndikufuna tikambe za chakudya chimene chimadyedwa pa banja panu

	Funso	Funso	Nambala
601.	Kodi pa masiku 30 apitawo m'nyumba mwanu munasowako chakudya chamtundu uliwonse chifukwa chosowa ndalama zogulira?	0 = Ayi → **Pitani pa 603**<br>1 = Inde	
602.	Izi zachitika kangati pa masiku 30 apitawa?	1 = Osati kwenikweni (kamodzi kapena kawiri)<br>2 = Nthawi zina (maulendo okwana atatu mpaka khumi)<br>3 = Kawirikawiri (kopitilira khumi)	
603.	Pa masiku 30 apitawa, kodi inu kapena wina pabanja panu adagonapo ndi njala?	0 = Ayi → **Pitani pa 605**	

		1 = Inde	
604.	Kodi zimenezi zidachitika kangati?	1 = Osati kwenikweni (kamodzi kapena kawiri)<br>2 = Nthawi zina (maulendo okwana atatu mpaka khumi)<br>3 = Kawirikawiri (kopitilira khumi)	
605.	Pa masiku 30 apitawa, kodi inu kapena ena pa banja panu adagonako ndi njala chifukwa chakuti chakudya chinali chochepa?	0 = Ayi → **Pitani pa 607**<br>1 = Inde	
606.	Zimenezi zachitika kangati pa masiku 30 apitawa?	1 = Osati kwenikweni (kamodzi kapena kawiri)<br>2 = Nthawi zina (maulendo okwana atatu mpaka khumi)<br>3 = Kawirikawiri (kopitilira khumi)	
607.	Tsopano ndikufunsani za mtundu wa chakudya umene inu kapena ena a pa banja lanu munadya dzulo masana ndi madzulo.<br><br>Sankhani zonse zimene zatchulidwa<br>A. CHILICHONSE [IKANI ZAKUDYA ZILIZONSE ZOPEZEKA MOSAVUTA, MONGA NSIMA], Buledi, mpunga, nudozi, mabisiketi,kapena chakudya chilichonse chochokera ku mapira, mawere, chimanga [IKANI ZAKUDYA ZILIZONSE ZOCHOKERA KU UFA]?<br><br>B.Mbatata, chilazi, chinangwa, zikhawo kapena zakudya zina zokumba pansi?<br><br>C. Ndiwo za masamba?<br><br>D. Zipatso?<br><br>E. Nyama ili yonse: yankhumba, yambuzi,yakalulu,, yakutchire, yankhuku, ya bakha, mbalame, chiwindi, impso, mtima, ziwalo zina<br><br>F. Mazira?<br><br>G.Nsomba zouma kapena zaziwisi?<br><br>H. Zakudya zina zilizonse zopangidwa kuchokera ku nyemba, ndozi, mtedza kapena nandolo?<br><br>I. Zakudya zochokera ku mkaka wa ziweto monga cheese, mkaka, yogati ndi zina?	A……………………………………|___| <br>B……………………………………|___| <br>C……………………………………|___| <br>D……………………………………|___| <br>E……………………………………|___| <br>F……………………………………|___|	

	J. Zakudya za mafuta monga mafuta ophikira, Majarini,butter?<br><br>K. Shuga kapena uchi?<br><br>L. Zakudya zina monga coffee, tiyi kapena zokometsera<br>M. Palibepo	G…………………………………|___| <br>H…………………………………|___| <br>I…………………………………|___| <br>J…………………………………|___| <br>K…………………………………|___| <br>L…………………………………|___| <br>M……………………………….I____I	

GAWO 7: Kupanga chiganizo ndi kudzidalira

Tsopano ndikufuna kuti tikambe za kupanga chiganizo ndi kudzidalira.

No.	Funso	Yankho	Nambala
701.	Mukapeza ndalama zanu, kodi ndi ndani amakupangirani maganizo pa ndalamazo mmene muzigwiritsire ntchito?	1 = Woyankha mafunso<br>2 = Mwamuna/bwenzi lanu<br>3 = Nonse (ndi mwamuma/chibwenzi kapena wina amene amakhala nawo pakhomopo)<br>4 = Munthu wadera ____________<br>5 = Woyankha mafunso samapeza ndalama	
702.	Kodi mukhoza kunena kuti ndalama zimene inu mumapeza ndi zoposa, zochepera kapena zofanana ndi zimene mwamuna/bwenzi lanu amapeza?	1 = Kuposa<br>2 = Kuchepera<br>3 = Mofanana<br>4 = Mwamuna samapeza ndalama<br>5 = Woyankhayu samapeza ndalama<br>6 = Palibe mwamuna/bwenzi<br>88 = DK/Refused<br>99 = zina _________________	if Q312=1
703.	Ndi ndani amene amapereka chiganizo momwe mwamuna/bwenzi lanu lingagwiritsire ntchito ndalama zake?	1 = Woyankha<br>2 = Mwamuna/bwenzi<br>3 = onse (ndi mwamuma/chibwenzi kapena wina amene amakhala nawo pakhomopo)<br>4 = Munthu wapadera ___________	if Q312=1

		5 = Palibe mwamuna /bwenzi	
704.	Kodi ndi ndani amene amapereka chiganizo pa katundu wofunika kugula pa banja panu?	1 = Woyankha<br>2 = Mwamuna/bwenzi<br>3 = onse (ndi mwamuma/chibwenzi kapena wina amene amakhala nawo pakhomopo)<br>4 = Munthu wapadera<br>____________	
705.	Kodi ndi ndani amene amapereka chiganizo cha umoyo wabwino pa kudzisamalira thanzi lanu?	1 = Woyankha<br>2 = Mwamuna/bwenzi<br>3 = onse (ndi mwamuma/chibwenzi kapena wina amene amakhala nawo pakhomopo)<br><br>4 = Munthu wapadera<br>____________	
706.	Kodi ndi ndani amene amapereka chiganizo cha umoyo wabwino pa kusamalira ana anu?	1 = Woyankha<br>2 = Mwamuna/bwenzi<br>3 = onse (ndi mwamuma/chibwenzi kapena wina amene amakhala nawo pakhomopo)<br><br>4 = Munthu wapadera<br>____________	If Q212 > 0
707.	Kodi ndi ndani amene amapereka chiganizo pa kuyendera abale kapena abwenzi anu?	1 = Woyankha<br>2 = Mwamuna/bwenzi<br>3 = onse (ndi mwamuma/chibwenzi kapena wina amene amakhala nawo pakhomopo)<br>4 = Munthu wapadera<br>____________	
	Kodi mumaloledwa kupita ku malo awa pa inu nokha, kapena pokhapokha mukaperekezedwa, kapena simuloledwa ndipango’no pomwe		
708.	Kumsika kukagula zinthu	0 = Saloledwa ndi pang’ono pomwe<br>1 = Pokhapokha akaperekezedwa<br>2 = Payekha	
709.	Kuchipatala	0= Saloledwa ndi pang’ono pomwe	

		1 = Pokhapokha akaperekezedwa<br>2 = Payekha	
710.	Kukacheza kwa anzanu	0= Saloledwa ndi pang'ono pomwe<br>1 = Pokhapokha akaperekezedwa<br>2 = Payekha	
711.	Ku tchalitchi/ kukachisi/ kumalo achipembedzo	0= Saloledwa ndi pang'ono pomwe<br>1 = Pokhapokha akaperekezedwa<br>2 = Payekha	
712.	Kutali ndi mzinda kukayendera abale/anzanu	0= Saloledwa ndi pang'ono pomwe<br>1 = Pokhapokha akaperekezedwa<br>2 = Payekha	

GAWO 8: Kukhala membala wa gulu ndi chithandizo cha m'magulu

No.	Funso	Yankho	Nambala
801.	Kodi pa miyezi isanu ndi umodzi yapitayi, ndi magulu ati pa magulu awa amene ali m'dera lanu? Nanga ndi magulu ati amene inu mumatengamo mbali?		
802.	Gulu la zaulimi/ziweto/usodzi	0 = Kulibe gululi<br>1 = Gulu alipo koma satenga nawo mbali<br>2= Woyankha mafunso amatenga nawo mbali mu gululi<br>88 = DK/Refuse	
803.	La za mabizinezi/ makalabu	0 = Kulibe gululi<br>1 = Gulu alipo koma satenga nawo mbali<br>2= Woyankha mafunso amatenga nawo mbali mu gululi<br>88 = DK/Refuse	
804.	Gulu la Zosunga ndi ku bwereketsana ndalama	0 = Kulibe gululi<br>1 = Gulu alipo koma satenga nawo mbali<br>2= Woyankha mafunso amatenga nawo mbali mu gululi<br>88 = DK/Refuse	
805.	La za ngongole (lokhazikika / losakhazikika)	0 = Kulibe gululi<br>1 = Gulu alipo koma satenga nawo mbali<br>2= Woyankha mafunso amatenga nawo mbali mu gululi<br>88 = DK/Refuse	
806.	La amayi	0 = Kulibe gululi<br>1 = Gulu alipo koma satenga nawo mbali	

		2= Woyankha mafunso amatenga nawo mbali mu gululi<br>88 = DK/Refuse	
807.	Gulu lokhuza za kachilombo ka HIV	00 = Kulibe gululi<br>1 = Gulu alipo koma satenga nawo mbali<br>2= Woyankha mafunso amatenga nawo mbali mu gululi<br>88 = DK/Refuse	
808.	Magulu ena othandiza anthu pa za umoyo wawo	0 = Kulibe gululi<br>1 = Gulu alipo koma satenga nawo mbali<br>2= Woyankha mafunso amatenga nawo mbali mu gululi<br>88 = DK/Refuse	
809.	La zipembedzo (lililonse)	0 = Kulibe gululi<br>1 = Gulu alipo koma satenga nawo mbali<br>2= Woyankha mafunso amatenga nawo mbali mu gululi<br>88 = DK/Refuse	
810.	La zandale	0 = Kulibe gululi<br>1 = Gulu alipo koma satenga nawo mbali<br>2= Woyankha mafunso amatenga nawo mbali mu gululi<br>88 = DK/Refuse	
811.	Pali magulu ena aku mudzi kuno amene mwatenga nawo mbali?	1 = Inde (fill in)<br>0 = Ayi	
	Pano ndukufuna tikambilane za chilimbikitso kuchokera kwa anthu amene mumacheza nawo.		
812.	Kodi pali munthu amene amakhalapo mukafuna chithandizo?	1 = Ndikukana kwambiri<br>2 = Ndikukana pang’ono<br>3 = ndili pakatikati<br>4 = Ndikuvomereza pang’ono<br>5 = Ndikuvomera kwambiri<br>88 = DK/Refuse	
813.	Kodi alipo munthu amene mumagana naye chisangalalo chanu ndi chisoni chanu?	1 = Ndikukana kwambiri<br>2 = Ndikukana pang’ono<br>3 = ndili pakatikati<br>4 = Ndikuvomereza pang’ono<br>5 = Ndikuvomera kwambiri<br>88 = DK/Refuse	
814.	Kodi banja lanu limayesetsa kumakuthandizani?	1 = Ndikukana kwambiri<br>2 = Ndikukana pang’ono<br>3 = ndili pakatikati<br>4 = Ndikuvomereza pang’ono<br>5 = Ndikuvomera kwambiri<br>88 = DK/Refuse	
815.	Kodi a pa banja panu amakuthandizani mukavutika m’maganizo?	1 = Ndikukana kwambiri<br>2 = Ndikukana pang’ono	

		3 = ndili pakatikati<br>4 = Ndikuvomereza pang'ono<br>5 = Ndikuvomera kwambiri<br>88 = DK/Refuse	
816.	Kodi muli ndi munthu wina wake amene amakutonthozani?	1 = Ndikukana kwambiri<br>2 = Ndikukana pang'ono<br>3 = ndili pakatikati<br>4 = Ndikuvomereza pang'ono<br>5 = Ndikuvomera kwambiri<br>88 = DK/Refuse	
817.	Kodi muli ndi anzanu amene amakuthandizani?	1 = Ndikukana kwambiri<br>2 = Ndikukana pang'ono<br>3 = ndili pakatikati<br>4 = Ndikuvomereza pang'ono<br>5 = Ndikuvomera kwambiri<br>88 = DK/Refuse	
818.	Kodi anzanu amene muli nawo mungawadalire zinthu zikalakwika?	1 = Ndikukana kwambiri<br>2 = Ndikukana pang'ono<br>3 = ndili pakatikati<br>4 = Ndikuvomereza pang'ono<br>5 = Ndikuvomera kwambiri<br>88 = DK/Refuse	
819.	Kodi mungathe kukamba za mavuto anu ndi a pa banja lanu?	1 = Ndikukana kwambiri<br>2 = Ndikukana pang'ono<br>3 = ndili pakatikati<br>4 = Ndikuvomereza pang'ono<br>5 = Ndikuvomera kwambiri<br>88 = DK/Refuse	
820.	Kodi muli ndi anzanu amene mungagawane nawo chimwemwe chanu ndi chisoni chanu?	1 = Ndikukana kwambiri<br>2 = Ndikukana pang'ono<br>3 = ndili pakatikati<br>4 = Ndikuvomereza pang'ono<br>5 = Ndikuvomera kwambiri<br>88 = DK/Refuse	
821.	Kodi alipo munthu m'moyo mwanu amene amasamalira malingaliro ndi zolakalaka zanu?	1 = Ndikukana kwambiri<br>2 = Ndikukana pang'ono<br>3 = ndili pakatikati<br>4 = Ndikuvomereza pang'ono<br>5 = Ndikuvomera kwambiri<br>88 = DK/Refuse	
822.	Kodi a pa banja lanu amafuna kumakuthandizani kupanga chiganizo? (decisions making)	1 = Ndikukana kwambiri<br>2 = Ndikukana pang'ono<br>3 = ndili pakatikati<br>4 = Ndikuvomereza pang'ono<br>5 = Ndikuvomera kwambiri<br>88 = DK/Refuse	

823.	Mungatiuzako mavuto anu pakati pa inu ndi anzanu?	1 = Ndikukana kwambiri<br>2 = Ndikukana pang'ono<br>3 = ndili pakatikati<br>4 = Ndikuvomereza pang'ono<br>5 = Ndikuvomera kwambiri<br>88 = DK/Refuse	

GAWO 9: Kumaliza mafunso (Endline Only)

Malagizo: Onetsani mayiyu buku lake la zandalama ndi kuunika zimene sizinapindule, zimene sizikuyenda bwino malinga ndimomwe zilili m'bukulo. Mukatha izo funsani mafunso otsatirawa:

901. Kodi mungafotokoze bwanji pa zomwe mwapeza posunga buku la zandalama pa miyezi 7 yapitayi?

902. Kodi maganizo anu pa zokhudza ndalama asintha kuchokera posunga bukuli? Ngati ndi choncho, asintha bwanji?

903. Kodi kagwiritsidwe ntchito kanu ka ndalama kasintha kuchokera mu kusunga buku la zandalama? Ngati ndi choncho, asintha bwanji?

904. Pamene tikupanga pulogalamu yatsopano yothandiza amayi woyembekezera ndi amayi amene angoyamba kubereka kumene za momwe angasamalire chuma chawo popititsa patsogolo kupewa kufalikira kwa kachirombo ka HIV kuchokera kwa mayi kupita kwa mwana, kodi ndi njira ziti zimene mukuganiza kuti zingakhale zothandiza kwambiri?

Kufunsitsa: Pa pulogalamu ya kusunga ndi kubwereketsa, funsitsani maganizo othandiza kupititsa patsogolo pulogalamuyi.

905. Kodi muli ndi maganizo omaliza kuti mugawane nafe pa momwe ndalama zanu zimalowera ndi kuchoka m'moyo wanu amene angakhale othandiza tikawadziwa?
