# Supplementary material for: Using financial diaries to understand the economic lives of HIV-positive pregnant women and new mothers in PMTCT in Zomba, Malawi
Source: PLoS One. 2021 Jul 30;16(7):e0252083. doi: 10.1371/journal.pone.0252083 (PMC8323884; doi:10.1371/journal.pone.0252083)
Supplement: S3 File — (PDF) [file pone.0252083.s003.pdf]

FHI 360

Financial Diary Debriefing Form
Version 2.0; May 29, 2018

Title: *Financial Diaries to Understand the Financial Needs of Pregnant Women and New Mothers in Zomba, Malawi*
Sponsor: *FHI 360 and USAID*
Address: *FHI 360, 359 Blackwell St, Suite 200, Durham, NC 27701 USA*

Notes for data collector:

- *Use this form for each debriefing interview with financial diary participants.*
- *Ask participants to disaggregate as much as possible. For example, if the participant went to the market on Monday and Wednesday, please include at least two entries for food instead of having one line for weekly food purchases. Disaggregate to specific food items if possible.*

Notes for programmer:

- The section in blue should be programmed as a group. During an interview, the data collector should be able to loop through the group as many times as necessary to capture all transactions. There will only be one group.

	**Questions**		**Responses**	**Relevance or Constraint**
**Int**		Interviewer ID	[fill-in]	only allow 100-300
**Today**		Date of interview	[calendar automated to mark system date, but can be modified by data collector]	
**Site**		Site/Location	0 = Likangala Health Centre (Rural)<br>1 = Pirimiti Community Hospital (Peri-urban)<br>2 = Matawale Health Centre (Urban)	
**PID1**		Participant PID	[fill-in]<br><br>*Note to programmer:*<br>*1001-1200 only valid if site=0*<br>*2001-2200 only valid if site=1*<br>*3001-3200 only valid if site=2*	see responses
**Week**		Week number	[fill in]	only allow 1-30
**Notes to Data Collector:**<br>**Remind the participant that you will be asking about all of her transactions over the course of the last week.**<br><br>**Give the specific dates/days for this week for her to keep in mind throughout the interview.**<br><br>**Briefly remind her of the different kinds of transactions she should report (cash, gifts, barters, debts/loans and savings).**				
	[Programmer: Start of new group]<br>**Read: Please tell me about the next transaction you would like to report.**			
	**Q1**	Based on the information provided by the participant, what type of transaction was this?	Choose only one:<br>0 = Cash transaction<br>1 = Barter<br>2 = Debt Repayment	

		A **cash transaction** is a transaction in which goods or services are exchanged for money.<br><br>A **barter** is a transaction in which goods or services are exchanged in both directions without money.<br><br>A **gift** is a transaction in which someone gives or receives a good, service or money and nothing is exchanged in return.<br><br>A **loan** is when someone gives or receives money (or sometimes a good or service) that must be paid back at a later time.<br><br>A **debt repayment** is when someone pays back or gets paid back for a loan.<br><br>A **savings** transaction is when money or assets are put into or taken out of a bank or savings group.<br><br>An **intra-household transfer** is when money is exchanged between household members without exchanging any goods or services. This differs from a gift in that it is for household management.<br><br>A **remittance** is when someone sends money to or receives money from far away.	3 = Loan<br>4 = Gift<br>5 = Savings<br>6 = Intra-household transfer<br>7 = Remittance	
	**Cash1**	Did you buy or sell something?	1 = Buy<br>2 = Sell	if Q1=0
	**buy_cash1**	What type of item/service did you buy?	*list of categories*	if Cash1=1
	**buy_cash2**	What did you buy?	*list of items based on category*	if Cash1=1
	**buy_cash3**	How many/much did you buy?<br>*If you aren't sure, take your best guess.*<br>*If she doesn't know, enter 8888.*	[integer fill in]	if Cash1=1
	**buy_cash4**	Quantity units	*list of units*	if Cash1=1
	**buy_cash5**	How much did you pay, in kwacha?	[integer fill-in]	if Cash1=1
	**sell_cash1**	What type of item/service did you sell?	*list of categories*	if Cash1=2
	**sell_cash2**	What did you sell?	*list of items based on category*	if Cash1=2
	**sell_cash3**	How many/much did you sell?<br>*If you aren't sure, take your best guess.*<br>*If she doesn't know, enter 8888.*	[integer fill in]	if Cash1=2

	**sell_cash4**	Quantity units	*list of units*	if Cash1=2
	**sell_cash5**	How much did you get paid, in kwacha?	[integer fill-in]	if Cash1=2
	*First, I'd like to talk about what you **gave** someone else in the barter.*			if Q1=1
	**Q2**	What type of item/service did you **give** to someone in the barter?	*List of categories*	if Q1=1
	**Q2a**	What specifically did you **give** to someone in the barter?	*List of items, based on response to Q2*	if Q1=1
	**Q2b**	How many/much did you **give**?<br>*If you aren't sure, take your best guess.*<br>*If she doesn't know, enter 888.*	[fill in number]	if Q1=1
	**Q2c**	Quantity units	*List of units*	if Q1=1
	**Q2d**	What is the estimated cash value of what you **gave** in kwacha?	[fill in number]	if Q1= 1
	*Now, I'd like to discuss what you **received** in the barter.*			if Q1= 1
	**Q2e**	What type of item/service did you **receive** in the barter?	*List of categories*	if Q1= 1
	**Q2f**	What specifically did you **receive** in the barter?	*List of items, based on response to Q2e*	if Q1= 1
	**Q2g**	How many/much did you **receive**?<br>*If you aren't sure, take your best guess.*<br>*If she doesn't know, enter 888.*	[fill in number]	if Q1= 1
	**Q2h**	Quantity units	*List of units*	if Q1= 1
	**Q2i**	What is the estimated cash value of what you **received** in kwacha?	[fill in number]	if Q1=1
	**Q3**	Was this transaction an inflow or an outflow?<br><br>An **inflow** is a transaction when the participant received something. This includes when she receives a gift, loan or someone pays back a debt owed to her. This also includes withdrawals from a bank account or share outs from a savings group.<br><br>An **outflow** is a transaction when gives something to someone else. This includes when she gives someone a gift or a loan or pays back a debt. This also includes deposits to a bank account or savings group.	0 = inflow<br>1 = outflow	if Q1=2, 3, 4, 5, 6 or 7
	**Q3a**	What type of item/service was this?	*List of categories*	if Q1=2, 3, 4, 5, 6 or 7
	**Q3b**	What was the specific item/service?	*List of items based on response to Q3a*	if Q1=2, 3, 4, 5, 6 or 7

	**Q3c**	How many/much?<br>*If you aren't sure, take your best guess. If she doesn't know, enter 888.*	[fill in number]	if Q1=2, 3, 4, 5, 6 or 7
	**Q3d**	Quantity units	*List of units*	if Q1=2, 3, 4, 5, 6 or 7
	**Q3e**	What is the (estimated) cash value in kwacha?<br>*If you aren't sure, take your best guess.*<br>*If she doesn't know, enter 888.*	[fill in number] Kwacha	if Q1=2, 3, 4, 5, 6 or 7<br><br>AND Q3a != Money
	**Q4**	Where did this transaction occur?	*List of locations*	
	**Q5**	Who was this transaction with?	*List of people*	
	**Q6**	What is the gender of the person the transaction was with?	0 = female<br>1 = male<br>2 = Not applicable<br>3 = Don't remember	
	**Q7**	What was the purpose of this transaction?	0 = Household/Personal<br>1 = Business<br>2 = Mixed	
	**Q8**	Was this transaction related to PMTCT or general health care?	0 = No, neither<br>1 = Yes, related to PMTCT care<br>2 = Yes, related to other health care	
	**You are about to finish entering data for this transaction. You will not be able to come back and edit this transaction. Swipe right if you are ready to continue to the next transaction. Swipe left to make edits.**			
**Please do a final probe for the following types of transactions: purchases, income, gifts, loans, debt repayments, barters, intrahousehold transfers, remittances, and savings that she has been involved in throughout the week. Double check to make sure you have captured all transactions. If you need to add another transaction, swipe left. If you are finished, swipe right.**				
**events**		Briefly describe any important events that happened this week. (e.g. weddings, travel, funeral, parties, etc)	[text fill-in]	
**PID2**		Re-enter the participant PIN	[fill-in]	Must match PID1
