# Supplementary material for: Using financial diaries to understand the economic lives of HIV-positive pregnant women and new mothers in PMTCT in Zomba, Malawi
Source: PLoS One. 2021 Jul 30;16(7):e0252083. doi: 10.1371/journal.pone.0252083 (PMC8323884; doi:10.1371/journal.pone.0252083)
Supplement: S4 File — (PDF) [file pone.0252083.s004.pdf]

FHI 360

Financial Diary Debriefing Form
Version 2.0; May 29, 2018

Title: *Financial Diaries to Understand the Financial Needs of Pregnant Women and New Mothers in Zomba, Malawi*
Sponsor: *FHI 360 and USAID*
Address: *FHI360, 359 Blackwell St, Suite 200, Durham, NC 27701 USA*

Notes for data collector:

- *Use this form for each debriefing interview with financial diary participants.*
- *Ask participants to disaggregate as much as possible. For example, if the participant went to the market on Monday and Wednesday, please include at least two entries for food instead of having one line for weekly food purchases. Disaggregate to specific food items if possible.*

Notes for programmer:

- The section in blue should be programmed as a group. During an interview, the data collector should be able to loop through the group as many times as necessary to capture all transactions. There will only be one group.

**Questions**		**Responses**	**Relevance or Constraint**
**Int**	Interviewer ID	[fill-in]	only allow 100-300
**Today**	Date of interview	[calendar automated to mark system date, but can be modified by data collector]	
**Site**	Site/Location	0 = Likangala Health Centre (Rural)<br>1 = Pirimiti Community Hospital (Peri-urban)<br>2 = Matawale Health Centre (Urban)	
**PID1**	Participant PID	[fill-in]<br><br>*Note to programmer:*<br>*1001-1200 only valid if site=0*<br>*2001-2200 only valid if site=1*<br>*3001-3200 only valid if site=2*	see responses
**Week**	Week number	[fill in]	only allow 1-30
**Notes to Data Collector:**<br>***Chidziwitso kwa enumerator:***<br><br>**Remind the participant that you will be asking about all of her transactions over the course of the last week.**<br>***Mukumbutseni ocheza nayeyo kuti mukhara mukucheza zo chitika zonse zokhudza ndalama zake ndi zinanso mu sabata yangothayi.***<br><br>**Give the specific dates/days for this week for her to keep in mind throughout the interview.**<br>***Mukumbutseni za masiku a sabata imeneyo kuti akhale akukumbukira mukecheza kwanu konse***<br><br>**Briefly remind her of the different kinds of transactions she should report (cash, gifts, barters, debts/loans and savings).**			

Mukumbutseninso za zochitika zosiyanasiyana zomwe akuyenera kucheza nanu , mwachitsanzo (za ndalama, mphatso, malonda osinthana popanda ndalama, ngongole yobwereka ndi kubwereketsa, ngakhalenso ndalama zo sunga)				
	[Programmer: Start of new group]<br>**Read: Please tell me about the next transaction you would like to report.**<br>***Werengani: Tandiuzeni zina zopatsidwa kapena kupereka zomwe zachitika mu sabata imeneyi***			
	**Q1**	Based on the information provided by the participant, what type of transaction was this?<br>***Kutengera nkhani zomwe mwapereka, inali inandi ndondomeko yantundu wanji?***<br><br>A **cash transaction** is a transaction in which goods or services are exchanged for money.<br>***Ndondomeko yogulila chinthu pogwiritsa ntchito ndalama***<br><br>A **barter** is a transaction in which goods or services are exchanged in both directions without money<br>***Ndondomeko yogula zinthu posinthana ndi katundu***<br><br>A **gift** is a transaction in which someone gives or receives a good, service or money and nothing is exchanged in return<br>**Ndondomeko yolandila thandizo mwaulele**<br><br>A **debt or a loan** is when someone gives or receives money (or sometimes a good or service) that must be paid back at a later time<br>**Ndondomeko yogula kapena kulandila thandizo pa ngongole**<br><br>A **savings** transaction is when money or assets are put into or taken out of a bank or savings group. **Ndondomeko yosunga chuma/ndalama**	Choose only one:<br>0 = Zokhudza ndalama<br>1 = Zosinthanitsa opanda ndalama<br>2 = Kubweza ngongole<br>3 = Kutenga ngongole<br>4 = Mphatso<br>5 = Kusunga ndalama<br>6 = Kupatsidwa ndalama kuchokera munyumba momwemo<br>7 = Kupatsidwa ndalama kuchokera kunja.	
	**Cash1**	Did you buy or sell something?<br>**Kodi munagura kapena kugulitsa chinachake?**	1 = Kugula<br>2 = Kugulitsa	if Q1=0
	**buy_cash1**	What type of item/service did you buy?<br>**Ndi katundu (zochitika) wanji amene munagura kapena kugulitsa?**	*list of categories*	if Cash1=1
	**buy_cash2**	What did you buy?<br>**Munagura chani?**	*list of items based on category*	if Cash1=1
	**buy_cash3**	How many/much did you buy?<br>**Munagura zochuluka(wochuluka) motani?**<br><br>*If you aren't sure, take your best guess.*<br>*If she doesn't know, enter 8888.*	[integer fill in]	if Cash1=1
	**buy_cash4**	Quantity units<br>**Muyezo**	*list of units*	if Cash1=1
	**buy_cash5**	How much did you pay, in kwacha?<br>**Kodi mudalipira zingati mu ma kwacha?**	[integer fill-in]	if Cash1=1

	**sell_cash1**	What type of item/service did you sell?<br>**Ndi katundu (zochitika) wanji amene munagulitsa?**	*list of categories*	if Cash1=2
	**sell_cash2**	What did you sell?<br>**Munagulitsa chani?**	*list of items based on category*	if Cash1=2
	**sell_cash3**	How many/much did you sell?<br>**Munagulitsa zochuluka(wochuluka) motani?**<br><br>*If you aren't sure, take your best guess.*<br>*If she doesn't know, enter 8888.*	[integer fill in]	if Cash1=2
	**sell_cash4**	Quantity units<br>**Muyezo**	*list of units*	if Cash1=2
	**sell_cash5**	How much did you get paid, in kwacha?<br>**Kodi adakulipirani zingati mu ma kwacha?**	[integer fill-in]	if Cash1=2
	*First, I'd like to talk about what you **gave** someone else in the barter.*			if Q1=1
	**Q2**	What type of item/service did you **give** to someone in the barter?<br>**Ndi ntundu wuti wa ntchito umene munapereka kwa munthu wina?**	*List of categories*	if Q1=1
	**Q2a**	What specifically did you **give** to someone in the barter?<br>**Munapereka chani kwenikweni kapena thandizo lanji ?**	*List of items, based on response to Q2*	if Q1=1
	**Q2b**	How many/much did you **give**?<br>**Munapereka zinthu zingati kapena thandizo lotani?**<br>*If you aren't sure, take your best guess.*<br>*If she doesn't know, enter 888.*	[fill in number]	if Q1=1
	**Q2c**	Quantity units<br>**Muyezo wake**	*List of units*	if Q1=1
	**Q2d**	What is the estimated cash value of what you **gave** in kwacha?<br>**Kuyerekeza ndi ndalama munapereka zingati mu makwacha?**	[fill in number]	if Q1= 1
	*Now, I'd like to discuss what you **received** in the barter.*			if Q1= 1
	**Q2e**	What type of item/service did you **receive** in the barter?<br>**Ndi chani kapena thandizo lanji lomwe munalandila kuchokera kwa munthu?**	*List of categories*	if Q1= 1
	**Q2f**	What specifically did you **receive** in the barter?<br>**Munalandila chani kapena thandizo lanji kuchokera kwa munthu wina?**	*List of items, based on response to Q2e*	if Q1= 1
	**Q2g**	How many/much did you **receive**?<br>**Ndi zinthu zingati kapena thandizo lotani limene munalandila?**<br><br>*If you aren't sure, take your best guess.*<br>*If she doesn't know, enter 888.*	[fill in number]	if Q1= 1
	**Q2h**	Quantity units<br>**Muyezo wake**	*List of units*	if Q1= 1

	**Q2i**	What is the estimated cash value of what you **received** in kwacha?<br>**Kuyerezeka ndi ndalama zinali zokwana ndalama zingati?**	[fill in number]	if Q1=1
	**Q3**	Was this transaction an inflow or an outflow?<br>**Kodi izi zinali zolowa kapena zotuluka?**<br><br>An **inflow** is a transaction when the participant received something. This includes when she receives a gift, loan or someone pays back a debt owed to her. This also includes withdrawals from a bank account or share outs from a savings group.<br>**Ndondomeko ya zinthu zolowa monga kupatsidwa mphatso, kulandila ngongole, kapena wina kukubwenzerani ngongole yanu. Kuphatikiza ndalama zomwe mwakatenga ku banki kapena ndalama zogawana ku banki m'khonde.**<br><br>An **outflow** is a transaction when gives something to someone else. This includes when she gives someone a gift or a loan or pays back a debt. This also includes deposits to a bank account or savings group.<br>**Ndondomeko ya zinthu zotuluka monga kupereka mphatso, kubwenza ngongole. Kuphatikizapo ndalama zomwe mwakasunga ku banki, kapena ku banki m'khonde.**	0 = Zolowa<br>1 = Zotuluka	if Q1=2, 3, 4, 5, 6 or 7
	**Q3a**	What type of item/service was this?<br>**munalandila chani?**	*List of categories*	if Q1=2, 3, 4, 5, 6 or 7
	**Q3b**	What was the specific item/service?<br>**ndi chani chenicheni chomwe munalandila/munapereka?**	*List of items based on response to Q3a*	if Q1=2, 3, 4, 5, 6 or 7
	**Q3c**	How many/much?<br>**Munalandila zinthu zingati kapena munapereka zinthu zingati?**<br><br>*If you aren't sure, take your best guess. If she doesn't know, enter 888.*	[fill in number]	if Q1=2, 3, 4, 5, 6 or 7
	**Q3d**	Quantity units<br>**Muyezo wake**	*List of units*	if Q1=2, 3, 4, 5, 6 or 7
	**Q3e**	What is the (estimated) cash value in kwacha?<br>**Tikayerekeza ndalama zimakwana zingati?**<br><br>*If you aren't sure, take your best guess.*<br>*If she doesn't know, enter 888.*	[fill in number] Kwacha	if Q1=2, 3, 4, 5, 6 or 7<br><br>AND Q3a != Money
	**Q4**	Where did this transaction occur?<br>**Ndondomekoyi inachitikila kuti?**	*List of locations*	
	**Q5**	Who was this transaction with?<br>**Ndondomekoyi inali ndi ndani?**	*List of people*	

	**Q6**	What is the gender of the person the transaction was with?<br>**Munthu anali ndi ndondomekoyi anali wa ntundu wanji?**	0 = Wamkazi<br>1 = Wamwamuna<br>2 = Not applicable<br>3 = Sakukumbukira	
	**Q7**	What was the purpose of this transaction?<br>**Chilinga cha ndondomekoyi Chinali chani?**	0 = Zapanyumba/zanga<br>1 = Geni<br>2 = Zambirimbiri	
	**Q8**	Was this transaction related to PMTCT or general health care?<br>**Kodi ndondomekoyi imakhudzana ndi kuteteza mayi posapatsira mwana wake kachilombo ka HIV kapena zaumoyo?**	0 = Ayi zonse<br>1 = Eya, zoteteza mwana kukachilombo<br>2 = Eya, chisamaliro cha za umoyo	
	**You are about to finish entering data for this transaction. You will not be able to come back and edit this transaction. Swipe right if you are ready to continue to the next transaction. Swipe left to make edits.**			
**Please do a final probe for the following types of transactions: purchases, income, gifts, loans, debt repayments, barters, intrahousehold transfers, remittances, and savings that she has been involved in throughout the week. Double check to make sure you have captured all transactions. If you need to add another transaction, swipe left. If you are finished, swipe right.**				
**events**		Briefly describe any important events that happened this week. (e.g. weddings, travel, funeral, parties, etc)<br>Tofotokozani mwachindunji zochitika zilizonse zomwe zachitika mu sabata imeneyi (mwachitsanzo, ukwati, ulendo, maliro)	[text fill-in]	
**PID2**		Re-enter the participant PIN	[fill-in]	Must match PID1
