# Supplementary material for: Using financial diaries to understand the economic lives of HIV-positive pregnant women and new mothers in PMTCT in Zomba, Malawi
Source: PLoS One. 2021 Jul 30;16(7):e0252083. doi: 10.1371/journal.pone.0252083 (PMC8323884; doi:10.1371/journal.pone.0252083)
Supplement: S5 File — (PDF) [file pone.0252083.s005.pdf]

FHI 360

In-Depth Interview Guide for Financial Diary Participants

Title:	*Financial Diaries to Understand the Financial Needs of Pregnant Women and New Mothers in Zomba, Malawi*
Sponsor:	*FHI 360 and USAID*
Address:	*FHI 360, 359 Blackwell St, Suite 200, Durham, NC 27701 USA*

Interview Guide

Section A: Experiences with PMTCT

To begin, I would like to learn about your experience receiving services to prevent mother-to-child transmission of HIV.

1. Tell me about how you became enrolled in PMTCT services.
    a. When were you diagnosed with HIV?
        i. Was this before or during your current/most recent pregnancy?
    b. At what point did you begin receiving PMTCT services (how many months pregnant/post-partum)?
    c. What initially motivated you to keep your PMTCT appointments and medication pick-ups?

2. How satisfied are you with the PMTCT services you receive at your local facility?
    a. Can you provide an example of what you find satisfying/dissatisfying about the experience?
    b. What, if anything, do you wish providers at your facility would do differently?

3. Who in your family or community supports you in receiving PMTCT services?
    - *Probe for examples of how people support her (emotionally, financially, provide child care, etc.)*
    - *Probe on support received from husband or partner, if any.*

    a. If no one, have you disclosed your status to your husband/partner or family?
        i. If not, why not?
        ii. If so, what happened when she disclosed to her husband or partner?

Section B: Financial Behaviors and PMTCT

Now I would like to talk about the cost of receiving PMTCT services, which can include the costs of any services you receive at the facility, any other cost related to getting to the facility and taking your medication on a regular basis, or lost opportunities for income.

4. What are the most common costs related to your PMTCT services?
    - *Probe for direct costs (i.e. facility services, transportation, child care)*
    - *Probe for indirect cost (i.e. lost wages or income from time spent going to the clinic)*

5. What are some factors that make it challenging to make your appointments or take your medication? [*Let individual respond without probing but, if necessary, provide examples such as not enough time, distance to the facility, needing to work or take care of other responsibilities, not sure PMTCT services are helping, etc.]*
    a. Specifically, how are each of these factors affecting your ability to stay in PMTCT care? Can you give me an example?

*[**Note:** For Q6-Q9, only ask about these specific topics if they were not addressed already under Q5.]*

6. How does food or lack of food affect whether you make your appointments or take your medication?
    - *If this is a barrier, probe for at least one example of how this is affecting their PMTCT care.*

7. How does having enough money or not having enough money affect whether you make your appointments or take your medication?
    - *If this is a barrier, probe for at least one example of how this is affecting their PMTCT care.*

8. How do your priorities and responsibilities at home or work affect whether you make your appointments or take your medication?
    - *If this is a barrier, probe for at least one example of how this is affecting their PMTCT care.*

9. Does travel or migration for business/work or other responsibilities affect whether you have regular access to clinical care in order to make your appointments or take your medication?
    - *If this is a barrier, probe for at least one example of how this is affecting their PMTCT care.*

10. Since you started PMTCT, have you experienced any new financial hardships that have made it difficult to make your appointments or take your medication? If so, please tell me about that. (If necessary, provide examples such as loss of job, harvest failure, family illness, etc.)
    a. What happened?
    b. At what point in your PMTCT care did this happen (how many months pregnant were you or how many months after your child was born)?
    c. How did this affect your ability to access PMTCT/ART services?

11. As we have been discussing, making all PMTCT-related appointments and medication pick-ups can be difficult for many reasons. Do you know about how many PMTCT appointments have you missed so far?
    Note: Question 11a will only be applicable to women who have had missed appointments. If the participant indicates that she has not missed any appointments, skip sub-question 11a and move to question 12.
    a. In thinking about the PMTCT appointments you have missed so far, about how many of them have been missed due, at least in part, to issues with money (i.e. not having transport money, not being able to take time away from income earning activities, not having food to take medications, etc.)
       [Facilitator: Use stones to assess proportion of all missed appointments due, at least in part, to financial reasons.]

12. Are there any specific services or support structures that help you pay for your PMTCT-related costs? If so, please tell me about them.
    - *Probe for types of services (i.e. cash transfers, financial incentives, social fund grants, financial support from family/friends, savings groups, income generating activities, etc.)*

13. Are there any personal strategies you use that help you remain in PMTCT care and pay for related costs? If so, please tell me about them.
    - *Probe for planning ahead for appointments and medication pick-ups*
    - *Probe for saving specifically for PMTCT related costs*

Section C: PMTCT Decision Making

Now, I want to learn more about the decisions you have had to make regarding continuing with PMTCT services.

14. Was there ever a time when you questioned or had concerns about continuing with PMTCT?
    a. If yes, did you stay in PMTCT care or drop out?

IF YES, and dropped out (15a)

Note to interviewer*: Only ask the following questions if respondent said she* <u>*dropped out of care*</u> *in Question 14 above.*
Ask ALL of the questions below

b. What were the main concerns? (Probes: economic or social pressures, family needs/wants, personal preferences, dissatisfaction with services, etc.)
c. How long after you enrolled in PMTCT did you start experiencing these concerns?
d. What was the most important deciding factor in leaving PMTCT care?
e. What were the trade-offs?
f. What additional support or services would have helped you to continue with PMTCT services?

IF YES, but stayed in (15b)

Note to interviewer*: Only ask the following questions if respondent said she did* <u>*question or had concerns*</u> *about continuing in PMTCT in Question 14 above, but* <u>*stayed in care*</u> *(Question 14a).*
Ask ALL of the questions below

What were the main concerns? (Probes: economic or social pressures, family needs/wants, personal preferences, dissatisfaction with services, etc.)

a. How long after you enrolled in PMTCT did you start experiencing these concerns?
b. What was the most important deciding factor in staying in PMTCT care?
c. What were the trade-offs?
d. What additional support or services would have made it easier to continue with PMTCT services?

IF NO, and stayed in (15c)

Note to interviewer*: Only ask the following question if respondent said she* <u>*did not have any concerns*</u> *in Question 14 above:*

a. Why is it important for you to continue with PMTCT services (probes: health of herself, health of her child, family pressure)?
b. What makes continuing with PMTCT services possible for you?

Section D: Community Perceptions of HIV

Now, I'd like to learn about your experience living with HIV in your community.

15. In general, what are the community perceptions or beliefs regarding people living with HIV?
    a. Is the perception generally positive, negative, or neutral?
    b. Is HIV stigmatized in your community?

16. What has your personal experience been like living with HIV in this community?
    We understand that everyone has a different experience, with some good and some bad, and we're interested in hearing examples of women's experiences.
    c. How have people in your community shown you acceptance?

d. How have people in your community discriminated against you?

17. What sort of support, if any, is provided to PLHIV in your community?
    - *Probe for formal and informal support*
