# Supplementary material for: Using financial diaries to understand the economic lives of HIV-positive pregnant women and new mothers in PMTCT in Zomba, Malawi
Source: PLoS One. 2021 Jul 30;16(7):e0252083. doi: 10.1371/journal.pone.0252083 (PMC8323884; doi:10.1371/journal.pone.0252083)
Supplement: S6 File — (PDF) [file pone.0252083.s006.pdf]

FHI 360

In-Depth Interview Guide for Financial Diary Participants in Financial Diaries Activity

Version 2.0, May 29, 2018

Title:	*Mabuku a zandalama pofuna kumvetsa nkhani za kusunga ndi kutsatira kulondoloza ndalama chuma za kwa azimayi oyembekezera komanso amene ali ndi ana akhanda mu boma la Zomba mu Malawi*
Sponsor:	*FHI 360 and USAID*
Address:	*FHI 360, 359 Blackwell St, Suite 200, Durham, NC 27701 USA*

Interview Guide

Section A: Experiences with PMTCT

To begin, I would like to learn about your experience receiving services to prevent mother-to-child transmission of HIV.

1. Tell me about how you became enrolled in PMTCT services.
   Ndiuzeni mmene munalembetsera pa nkhani za mayi kuteteza mwana kukachirombo ka HIV
   - **a.** When were you diagnosed with HIV? **Munapezeka nawo liti kachiromboka?**
     - **i.** Was this before or during your current/most recent pregnancy? **Munali musanatenge mimba kapena nthawi yomwe munali ndi mimba yaposachedwayi?**
   - **b.** At what point did you begin receiving PMTCT services (how many months pregnant/post-partum)? **Munayamba kulandila thandizo pa miyezi ingati?**
   - **c.** What made you decide to initially motivated you to keep your PMTCT appointments and medication pick-ups **Chinakupangitsani ndi chani kuti muyambe komanso kumakatenga mankhwara?**

2. How satisfied are you with the PMTCT services you receive at your local facility? **Ndinu okhotitsidwa bwanji pa thandizo lomwe mumalandila pachipatala chakumudzi chino?**
   - **a.** Can you provide an example of what you find satisfying/dissatisfying about the experience? **Mungapereke chitsanzo pazimene mmakhutitsidwa kapena kusakhutitsidwa nawo kutengera ndi mmene mukudziwila?**
   - b. What, if anything, do you wish providers at your facility would do differently? **Ndi chani chimene mungakonde opereka thandizo atapanga mosiyana?**

3. Who in your family or community supports you in receiving PMTCT services? **Polandila thandizoli amakuthandizilani ndindani wa m'banja mwanu kapena kudera kwanu?**
   - *Probe for examples of how people support her (emotionally, financially, provide child care, etc.)*
   - *Probe on support received from husband or partner, if any.*

a. If no one, have you disclosed your status to your husband/partner or family? **Ngati palibe munawuza amuna anu/ wokondedwa wanu kapena achibale za mmene mulili?**
    i. If not, why not? **Ngati ayi chifukwa chani?**
    ii. If so, what happened when she disclosed to her husband or partner? **Ngati eya, chinachitika ndi chani mutawawuza amuna wanu kapena wokondedwa wanu.**

Section B: Financial Behaviors and PMTCT

Now I would like to talk about the cost of receiving PMTCT services, which can include the costs of any services you receive at the facility, any other cost related to getting to the facility and taking your medication on a regular basis, or lost opportunities for income. **Tsopano tikambilana za mmene mmalandilila thandizo, zimene ziphatikizire thandizo lililonse limene mmalandila pachipatala kapena chilichonse chokhudzana ndi chipatala ndikutenga mankhwala nthawi ndi nthawi kapena kutaya mwawi wandalama.**

4. What are the most common costs related to your PMTCT services? **Ndi zolowa ziti zomwe zimakulowani mthumba poteteza mwana ku kachilombo ka EDZI.**
    - *Probe for direct costs (i.e. facility services, transportation, child care)*
    - *Probe for indirect cost (i.e. lost wages or income from time spent going to the clinic)*

5. What are some factors that make it challenging to make your appointments or take your medication? [*Let individual respond without probing but, if necessary, provide examples such as not enough time, distance to the facility, needing to work or take care of other responsibilities, not sure PMTCT services are helping, etc.]* ***ndi zinthu ziti zomwe zinali zovuta kupanga ndondomeko yokumana ndi dokotala kapena kulandila mankhwala?***
    a. Specifically, how are each of these factors affecting your ability to stay in PMTCT care? Can you give me an example? **mwachindunji, zakhudza bwanji kukhala kwanu la thandizo limeneli?**

*[**Note:** For Q6-Q9, only ask about these specific topics if they were not addressed already under Q5.]*

6. How does food or lack of food affect whether you make your appointments or take your medication? **Kusowa kwa chakudya kwakhudza bwanji ndi ndondomeko kapena kulandila mankhwala?**
    - *If this is a barrier, probe for at least one example of how this is affecting their PMTCT care.*

7. How does having enough money or not having enough money affect whether you make your appointments or take your medication? **Kodi kukhala ndi ndalama kapena kukhala opanda ndalama zokwanila kwadzudzana bwanji ndi ndondomeko yokumana ndi dokotala kapena kulandila thandizo lanu la mankhwala?**

- *If this is a barrier, probe for at least one example of how this is affecting their PMTCT care.*

8. How do your priorities and responsibilities at home or work affect whether you make your appointments or take your medication? **Kodi zofuna kapena udindo wanu pakhomo kapena ku ntchito zakhudza bwanji ndondomeko yowonana ndi dokotala kapena kulandila mankhwala?**
    - *If this is a barrier, probe for at least one example of how this is affecting their PMTCT care.*

9. Does travel or migration for business/work or other responsibilities affect whether you have regular access to clinical care in order to make your appointments or take your medication? **Kodi kuyenda maulendo abusiness kapena ntchito ndi maudindo ena akhudzana ndi ndondomeko zowonana ndi dokotala kapena kulandila mankhwala?**
    - *If this is a barrier, probe for at least one example of how this is affecting their PMTCT care.*

10. Since you started PMTCT, have you experienced any new financial hardships that have made it difficult to make your appointments or take your medication? If so, please tell me about that. (If necessary, provide examples such as loss of job, harvest failure, family illness, etc.)
chiyambileni pulogalamu ya PMTCT munayamba mwakhalapo ndi vulo lazachuma kuti mmalephela kukawonanana ndi dokotala komaso kukalandizo thandizo lamankhwala? Ngati ndi choncho ndiuzeni. (popeleka chitsanzo ngati kutha kwa ntchito, kukanika kwa zokolola, matenda)
    a. What happened? **Chinachitika ndi chani?**
    b. At what point in your PMTCT care did this happen (how many months pregnant were you or how many months after your child was born)? **Munali ndi mimba ya miyezi ingati kapena mwana wanu anali ndi miyezi ingati atabadwa pamene izi zimachitika?**
    c. How did this affect your ability to access PMTCT/ART services? **Zinakukhudzani bwanji zimenezo pokapeza thandizo la mankhwala?**

11. As we have been discussing, making all PMTCT-related appointments and medication pick-ups can be difficult for many reasons. Do you know about how many PMTCT appointments have you missed so far? **Monga takhara tikukambirana, kulondoloza ma ulendo onse omwe mumayenera kupita ku chipatala pa zokhudza kuteteza mwana ndi kukatenga mankhwala ndikovuta pa zifukwa zambiri. Kodi mungadziwe kuti mudadumphitsapo kangati kufikira pono?**

Note: Question 11a will only be applicable to women who have had missed appointments. If the participant indicates that she has not missed any appointments, skip sub-question 11a and move to question 12. **Ngati sadaduphitsepo, musafunse funso 11a, pitani ku funso 12.**

11a. In thinking about the PMTCT appointments you have missed so far, about how many of them have been missed due, at least in part, to issues with money (i.e. not having transport money, not being able to take time away from income earning activities, not having food to take medications, etc.)
kungoganizila maulendo amene munakanika kukawonana ndi dokotala, ndi maulendo angati munakanika Kamba kavuto la ndalama(kukhala opanda ndamala yoyendera, kukanika kusiya ntchito zomwe zimawapatsa ndalama,kusowa chakudya kuti amwe mankhwala)
[Facilitator: Use pie-chart visual to assess proportion of all missed appointments due, at least in part, to financial reasons.]

12. Are there any specific services or support structures that help you pay for your PMTCT-related costs? If so, please tell me about them.
Pali thandizo kapena njira zomwe zimakuthandizani kulipila zonse zokhudzana ndi PMTCT?
    - *Probe for types of services (i.e. cash transfers, financial incentives, social fund grants, financial support from family/friends, savings groups, income generating activities, etc.)*

13. Are there any personal strategies you use that help you remain in PMTCT care and pay for related costs? If so, please tell me about them.
Pali njira zomwe mmagwiritsa ntchito kuti mulipile ndi cholinga choti mukhalebe mu pulogalamu ya PMTCT? Chonde ndiuzeni
    - *Probe for planning ahead for appointments and medication pick-ups*
    - *Probe for saving specifically for PMTCT related costs*

Section C: PMTCT Decision Making
Now, I want to learn more about the decisions you have had to make regarding continuing with PMTCT services.
Tsopano ndikufuna ndiphunzire nawo za mmene mmapangira chiganizo zokhuzana ndi thandizo la PMTCT

14. Was there ever a time when you questioned or had concerns about continuing with PMTCT?
Inalipo nthawi yomwe mudazifunza kapena kukhala ndi nkhwaza zakupitiliza pulogalamu ya PMTCT?
    a. If yes, did you stay in PMTCT care or drop out?
    Ngati eya, munapitiliza kapena munasiya?

IF YES, and dropped out (15a)

Note to interviewer*: Only ask the following questions if respondent said she dropped out of care in Question 14 above.*
Ask ALL of the questions below

a. What were the main concerns? (Probes: economic or social pressures, family needs/wants, personal preferences, dissatisfaction with services, etc.)
   Nkhawa zanu zeni zeni zinali ziti?

b. How long after you enrolled in PMTCT did you start experiencing these concerns?
   Panapita nthawi yaitali bwanji kuti muyambe kuda nkhawa ka zimenezo?

c. What was the most important deciding factor in leaving PMTCT care?
   Ndi mfundo iti yayikulu yomwe mudaganiza zokhalabe mu pulogalamuyu?

d. What were the trade-offs?
   Kodi ndi kusinthana kotani komwe kwachitika pokhudzana ndi PMTCT?

e. What additional support or services would have helped you to continue with PMTCT services?
   Panaliso ma thandizo ena owonjezera amene anali apafupi kupitiliza pulogalamu ya PMTCT?

IF YES, but stayed in (15b)

Note to interviewer*: Only ask the following questions if respondent said she did question or had concerns about continuing in PMTCT in Question 14 above, but stayed in care (Question 14a).*
Ask ALL of the questions below

a. What were the main concerns? (Probes: economic or social pressures, family needs/wants, personal preferences, dissatisfaction with services, etc.)
   Nkhawa zanu zeni zeni zinali ziti?

b. How long after you enrolled in PMTCT did you start experiencing these concerns?
   Papita nthawi yaitali bwanji chiyambileni kuda nkhawa ka zimenezo?

c. What was the most important deciding factor in staying in PMTCT care?
   Ndi mfundo iti yayikulu yomwe mudaganiza zokhalabe mu pulogalamuyu?

d. What were the trade-offs?
   Kodi ndi kusinthana kotani komwe kwachitika pokhudzana ndi PMTCT?

e. What additional support or services would have made it easier to continue with PMTCT services?
   Panaliso ma thandizo ena owonjezera amene anali apafupi kupitiliza pulogalamu ya PMTCT?

IF NO, and stayed in (15c)

Note to interviewer: *Only ask the following question if respondent said she* *did not have any concerns* *in Question 14 above:*

a. Why is it important for you to continue with PMTCT services (probes: health of herself, health of her child, family pressure)?
   Chifukwa chani mukupitiliza kukhalabe mu pulogalamu ya PMTCT?

b. What makes continuing with PMTCT services possible for you?
   Ndichani chomwe chinakupangitsani kuti mupitilize/mukhalabe mu pulogalamu ya PMTCT?

Section D: Community Perceptions of HIV

Now, I'd like to learn about your experience living with HIV in your community.

Tsopano ndiphunzira nawo za mmene mwakhalila m'dera lano lino pamene muli ndi kachilombo koyambitsa matenda a Edzi.

15. In general, what are the community perceptions or beliefs regarding people living with HIV?
Kodi anthu akudera kuno maonedwe ndi zikhulupililo zawo ndizotani pokhudzana ndi anthu amene ali ndi kachilombo koyambitsa matenda a edzi?

a. Is the perception generally positive, negative, or neutral?
   Maonedwe awo ngothandiza kapena ngosathandiza kapena ali pakatikati?
b. Is HIV stigmatized in your community?
   kodi HIV imasalidwa kudera kwanu?

16. What has your personal experience been like living with HIV in this community?
Kukhala kwanu m'dera lanu mungatifotokozere zotani zokhudza HIV?
We understand that everyone has a different experience, with some good and some bad, and we're interested in hearing examples of women's experiences.
Timamva kuti aliyense ali ndi zimene wadutsamo, zabwino kapena zoipa ndipo tikufunitsitsa zitsanzo zomwe azimayi adutsamo pokhudzana ndi HIV

a. How have people in your community shown you acceptance?
   Mmene anthu akudera kwanu anakulandililani?
b. How have people in your community discriminated against you?
   Mmene anthu akudera kwanu amakusiyanitsirani ndi anthu ena?

17. What sort of support, if any, is provided to PLHIV in your community?
Pali thandizo lanji lomwe timaperekedwa kwa anthu omwe ali ndi kachilomo kudera kwanu, ngati lilipo?

- *Probe for formal and informal support*
